# Supplementary material for: Enzymatic one-step ring contraction for quinolone biosynthesis
Source: Nat Commun. 2018 Jul 19;9:2826. doi: 10.1038/s41467-018-05221-5 (PMC6053404; doi:10.1038/s41467-018-05221-5)
Supplement: Supplementary file 1 — Supplementary Information [file 41467_2018_5221_MOESM1_ESM.pdf]

Supplementary Information for

Enzymatic one-step ring contraction for quinolone biosynthesis

Shinji Kishimoto,[1] Kodai Hara,[1] Hiroshi Hashimoto,[1] Yuichiro Hirayama,[1] Pier Alexandre Champagne,[2] Kendall N. Houk,[2,3] Yi Tang,[2,3] Kenji Watanabe,[1*]

[1]Department of Pharmaceutical Sciences, University of Shizuoka, Shizuoka 422-8526, Japan
[2]Department of Chemistry and Biochemistry and [3] Department of Chemical and Biomolecular Engineering, University of California, Los Angeles, California 90095, United States

***Correspondence e-mail: kenji55@u-shizuoka-ken.ac.jp**

1. Supplementary Figures

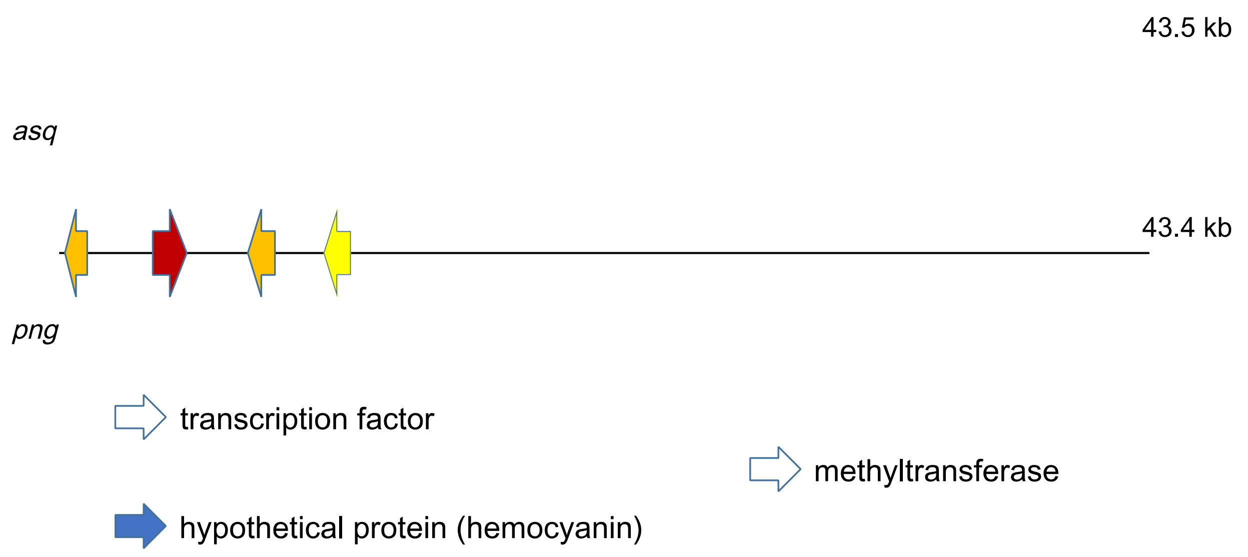

Supplementary Figure 1. Aspoquinolone biosynthetic gene cluster (*asq*) in *Aspergillus nidulans* A1149 and penigequinolone biosynthetic gene cluster (*png*) in *Penicillium* sp. FKI-2140.

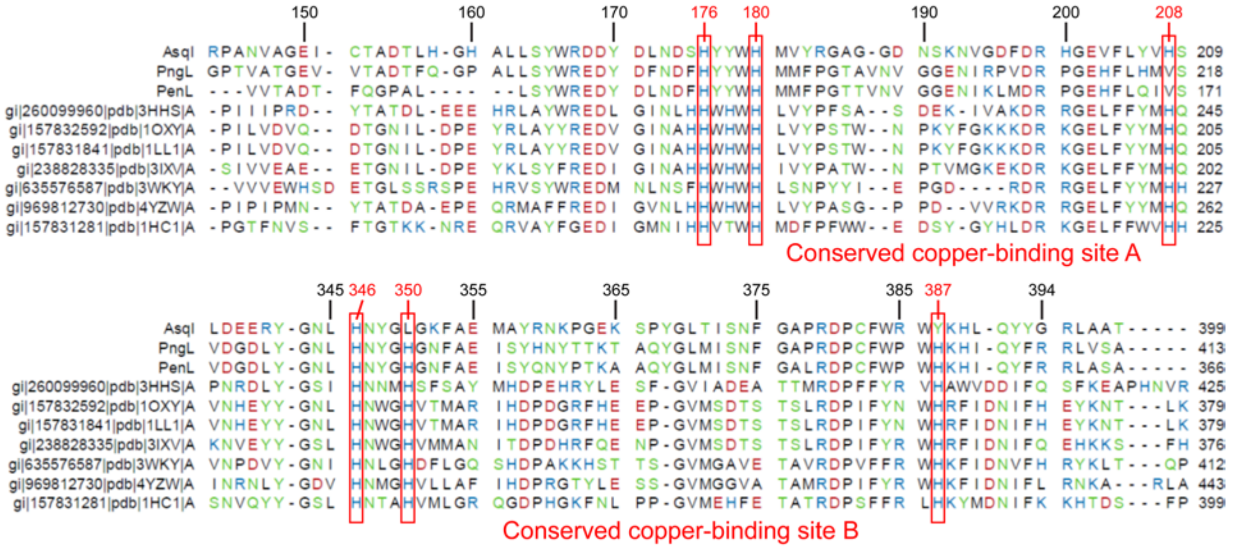

Supplementary Figure 2. The amino acid sequence alignment of the conserved metal-binding sites A and B of AsqI, PenL[1] and PngL with representative structurally known members of the hemocyanin M family (Pfam ID PF00372)[2]. The alignment was prepared using the program Clustal Omega[3]. Each of the conserved three histidine residues is indicated by a red box. The sequence numbering at to top is that of AsqI. 3HHS: *Manduca sexta* (tobacco hornworm) prophenoloxidase[4]; 1OXY: oxygenated *Limulus polyphemus* (Atlantic horseshoe crab) hemocyanin subunit II[5]; 1LL1: hydroxo-bridge met-form *Limulus polyphemus* hemocyanin subunit II[6]; 3IXV: *Androctonus australis* (scorpion) hemocyanin; 3WKY[7]: *Marsupenaeus japonicus* (Japanese tiger prawn) hemolymph-type prophenoloxidase[8]; 4YZW: Anopheles gambiae (African malaria mosquito) prophenoloxidase[9]; and 1HC1: *Panulirus interruptus* (California spiny lobster) haemocyanin[10].

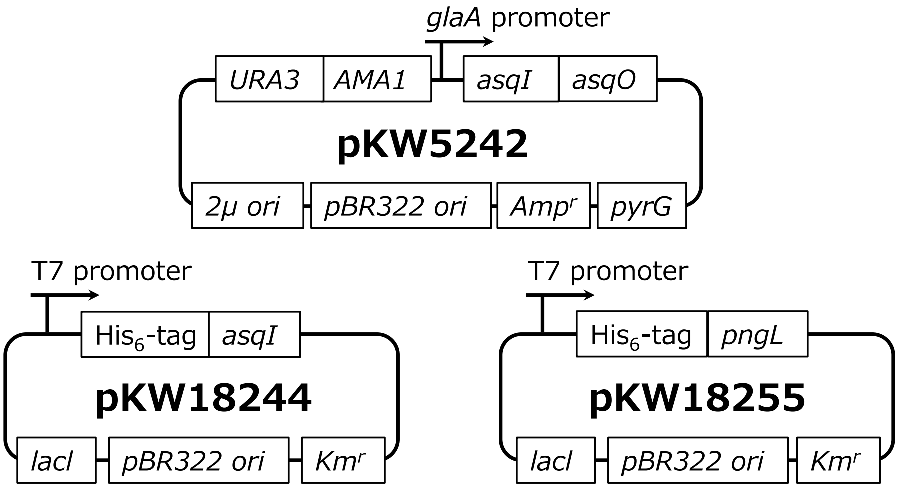

Supplementary Figure 3. Maps of plasmid pKW5242 for the cloning of *asqI* and pKW18244 and pKW18255 for the production of AsqI and PngL in *Escherichia coli*.

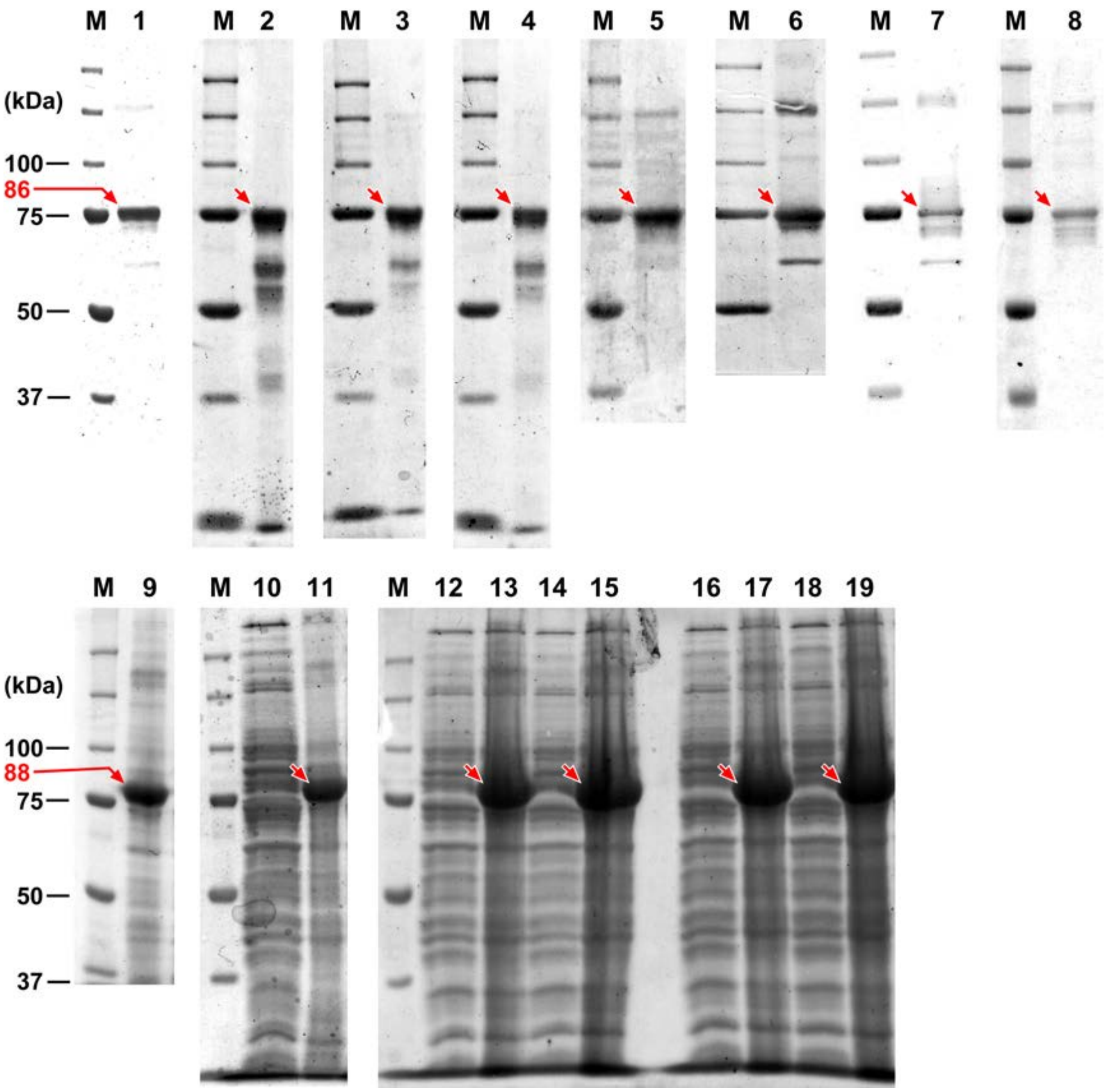

Supplementary Figure 4. SDS–PAGE analysis of the purified AsqI (86 kDa), partially purified PngL (88 kDa) and mutated enzymes stained with CBB. Lane M: molecular weight marker. Top panel: AsqI and its mutants. Lanes 1: Wild type; 2: His176Ala mutant; 3: His180Ala mutant; 4: His208Ala mutant; 5: His346Ala mutant; 6: Arg184Ala mutant; 7: Asp322Leu mutant; 8: Asn347Leu mutant. Bottom panel: PngL and its mutants. L9: Wild type; 10: His184Ala mutant soluble fraction; 11: His184Ala mutant insoluble fraction; 12: His188Ala mutant soluble fraction; 13: His188Ala mutant insoluble fraction; 14: His360Ala mutant soluble fraction; 15: His360Ala mutant insoluble fraction; 16: His364Ala mutant soluble fraction; 17: His364Ala mutant insoluble fraction; 18: His401Ala mutant soluble fraction; 19: His401Ala mutant insoluble fraction.

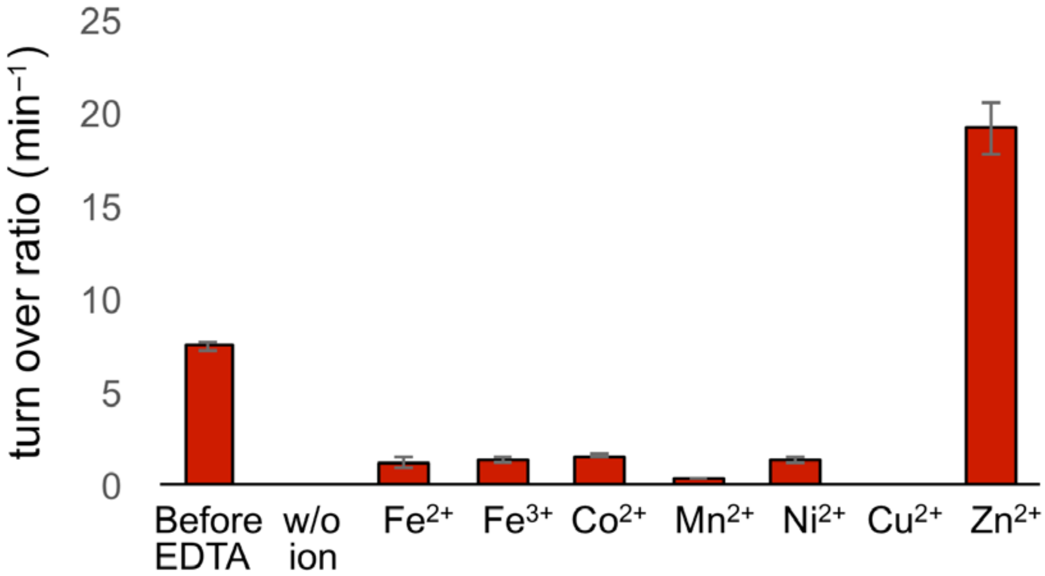

Supplementary Figure 5. Metal requirement of the activity for AsqI. AsqI was dialyzed against EDTA buffer (100 μM EDTA, 100 mM Tris-HCl, 100 mM NaCl, pH 7.4) for 3 h at 4 °C, and was dialyzed against 10 mM Tris buffer (10 mM Tris-HCl, 100 mM NaCl, pH 7.4) for 3 h at 4 °C. After dialysis, AsqI was concentrated to 40 μM using a 30K Amicon Ultra centrifugal concentrator (Millipore). Reaction mixtures containing 2 μM of EDTA-treated AsqI, 100 μM of each of the metal ions Fe^{2+}, Fe^{3+}, Co^{2+}, Mn^{2+}, Ni^{2+}, Cu^{2+} or Zn^{2+}, and 0.8 mM of **5** were incubated for 3 minutes at 30 °C. The reaction was quenched with EtOAc containing 10 μM of anthraquinone as an internal standard and analyzed as described in the **Methods** section. The measurement for each metal is a mean of triplicate measurements. The standard deviation is given in the plot as an error bar at the top of the bar.

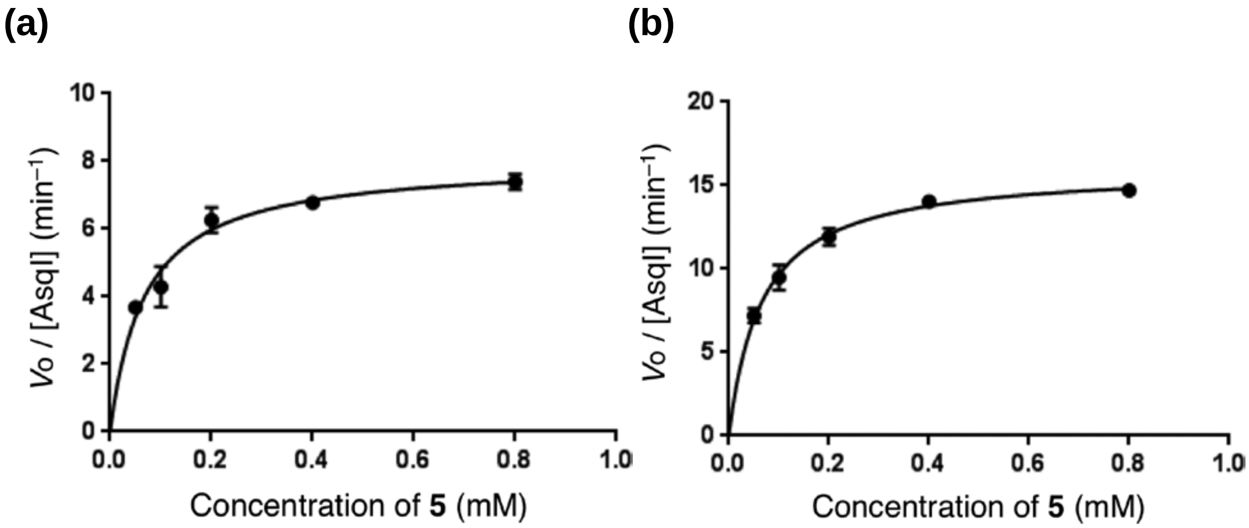

Supplementary Figure 6. Michaelis–Menten kinetics for the formation of viridicatin **6** from the substrate (–)-cyclopenin **5** catalyzed by AsqI in the (a) absence or (b) presence of 30 µM $ZnCl_2$. Each data point is a mean of triplicate measurements. The standard deviation is given in the plot as an error bar at each data point. All of the kinetic analyses were conducted using the method described in the **Methods** section of the main text.

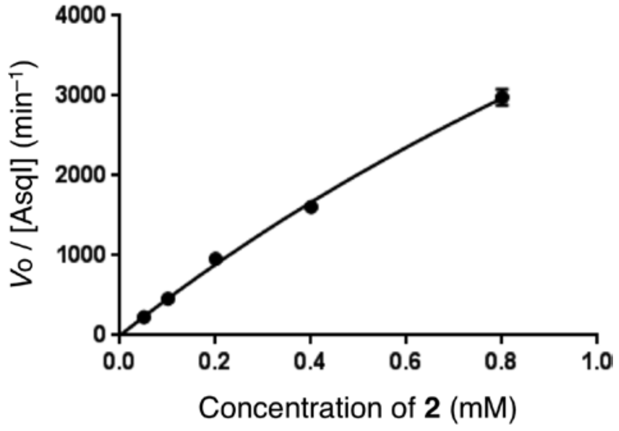

Supplementary Figure 7. Michaelis–Menten kinetics for the formation of 4'-methoxyviridicatin **3** from the substrate (–)-4'-methoxycyclopenin **2** catalyzed by AsqI. Each data point is a mean of triplicate measurements. The standard deviation is given in the plot as an error bar at each data point. All of the kinetic analyses were conducted using the method described in the **Methods** section of the main text.

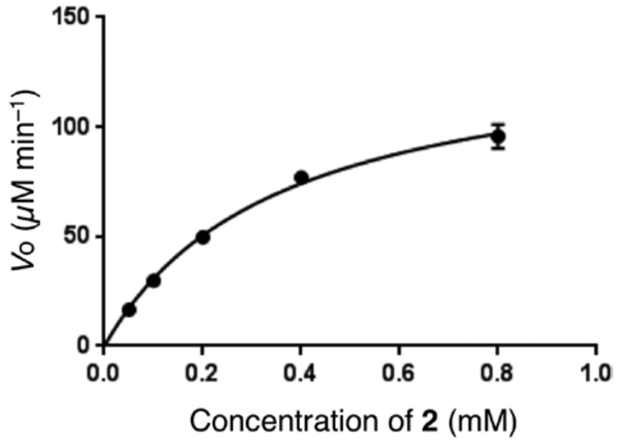

Supplementary Figure 8. Michaelis–Menten kinetics for the formation of **3** from the substrate **2** catalyzed by PngL. Each data point is a mean of triplicate measurements. The standard deviation is given in the plot as an error bar at each data point. All of the kinetic analyses were conducted using the method described in the **Methods** section of the main text.

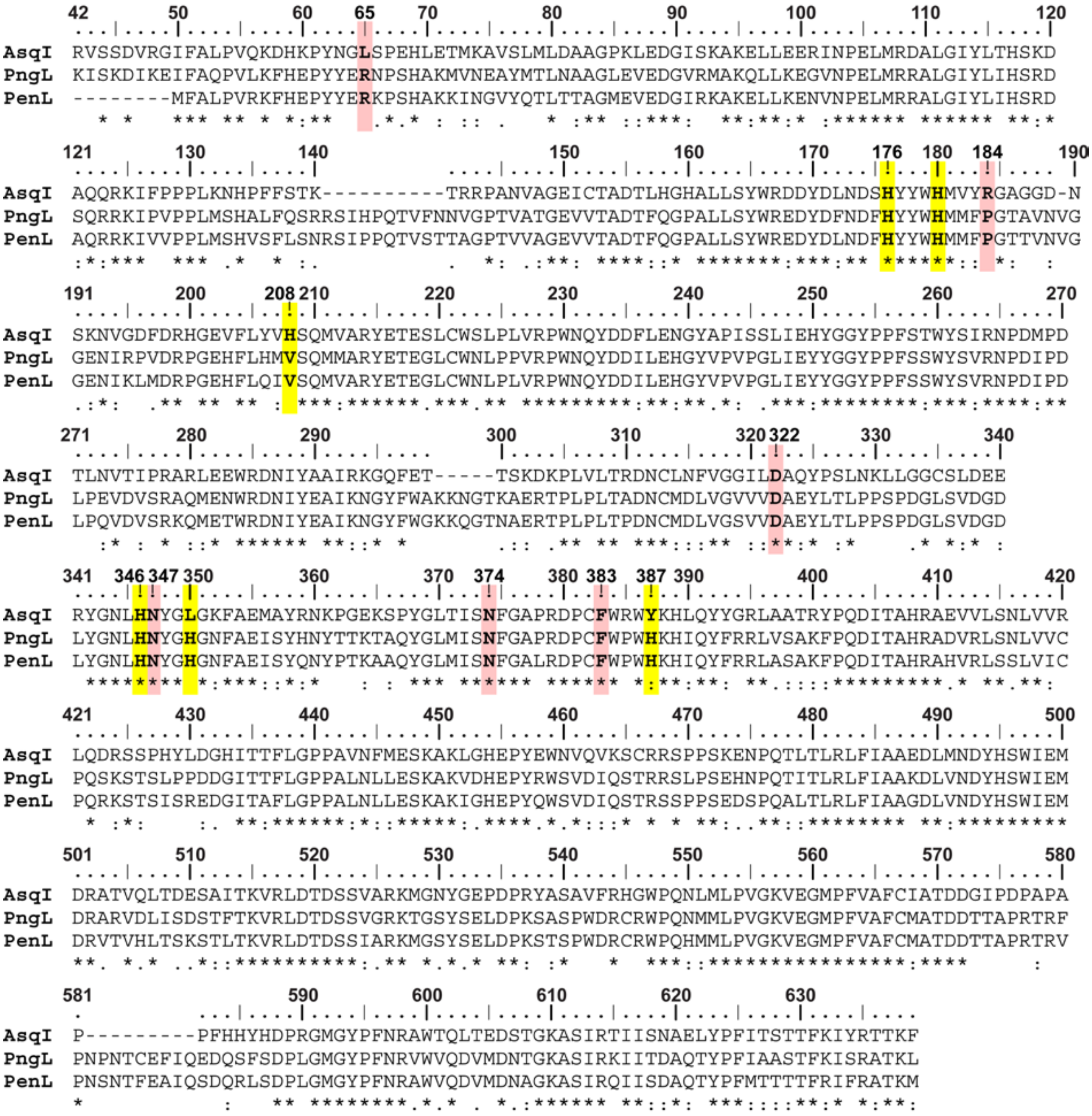

Supplementary Figure 9. Amino acid sequence alignment of the cyclopenase-type enzymes AsqI, PngL and PenL[1]. The alignment was prepared using the program Clustal Omega[3]. Conserved and strongly similar residues are denoted by asterisks and colons, respectively. The residues corresponding to the conserved metal-binding residues in hemocyanins are highlighted in yellow. Residues in AsqI near the metal-binding site show in **Fig. 3a** and their corresponding residues in PngL and PenL are highlighted in pink. Residue numbering is that of AsqI.

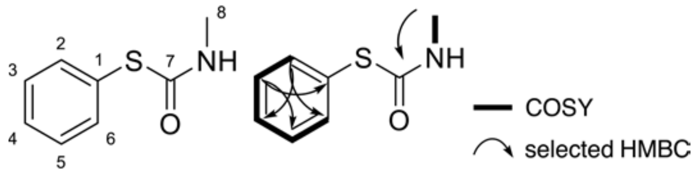

Supplementary Figure 10. Chemical structure of **9** and relevant two-dimensional nuclear magnetic resonances.

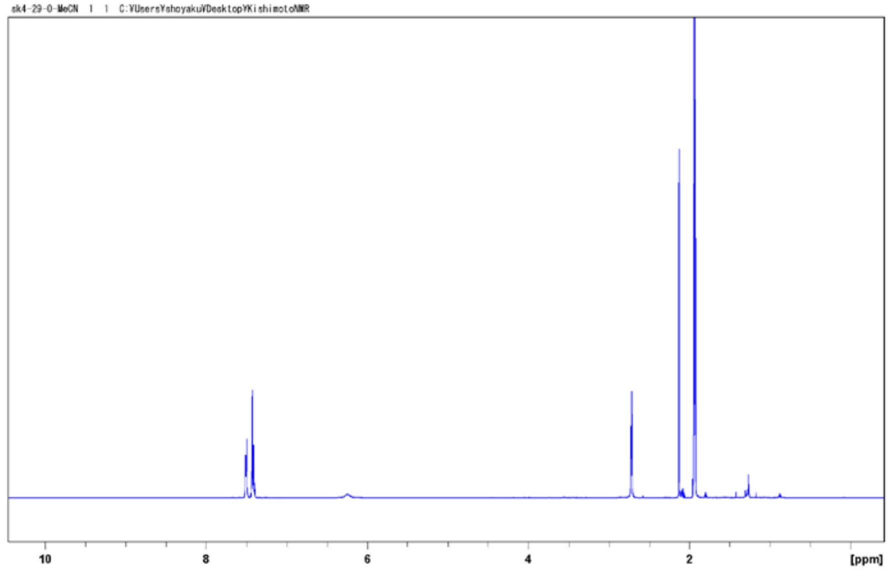

Supplementary Figure 11. ^{1}H NMR (500 MHz) spectrum of **9** in CD_3CN.

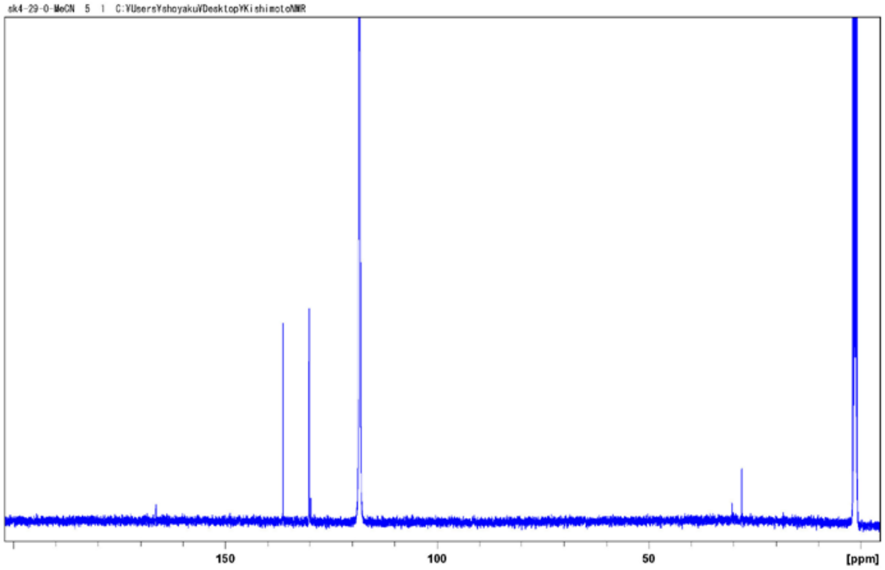

Supplementary Figure 12. ^{13}C NMR spectrum of **9** in CD_3CN (125 MHz).

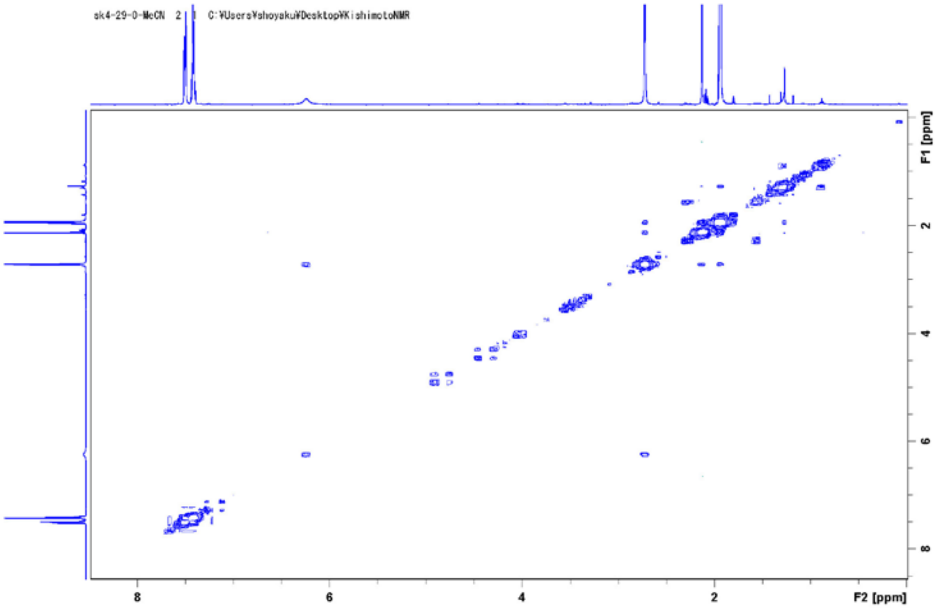

Supplementary Figure 13. COSY spectrum of **9** in CD_3CN (500 MHz).

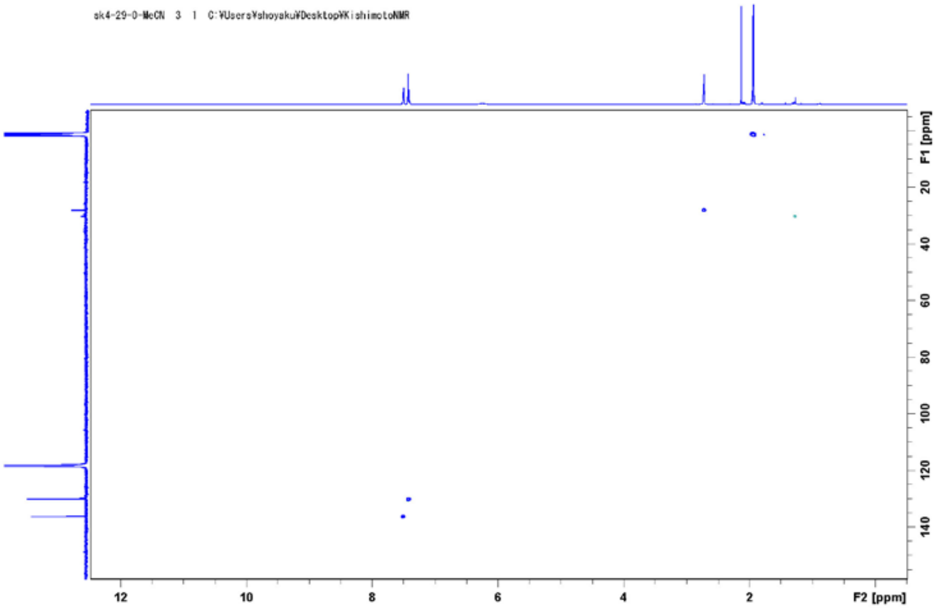

Supplementary Figure 14. HSQC spectrum of **9** in CD_3CN (500 MHz).

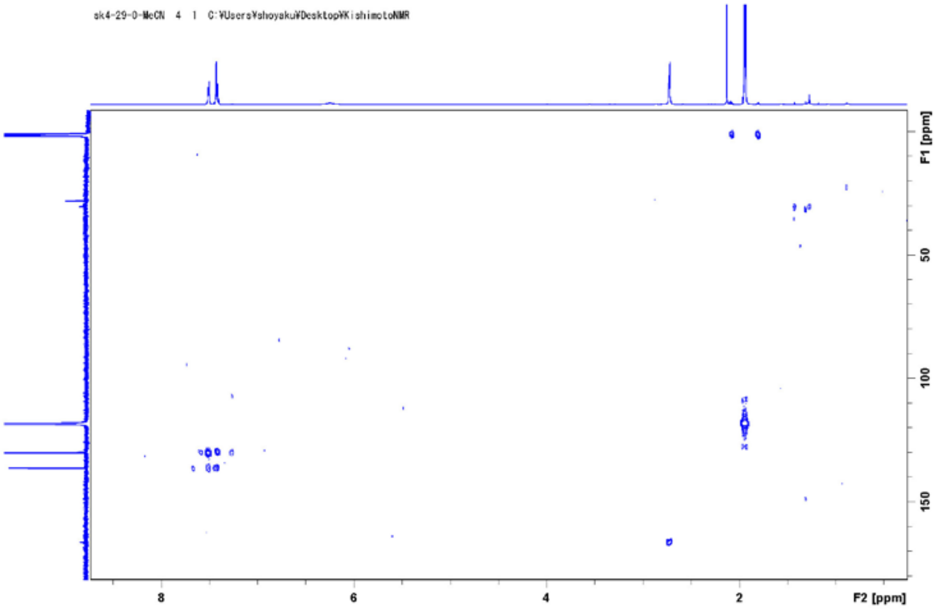

Supplementary Figure 15. HMBC spectrum of **9** in CD_3CN (500 MHz).

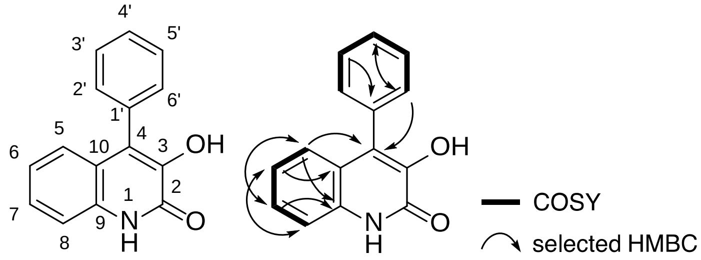

Supplementary Figure 16. Chemical structure of **6** and relevant two-dimensional nuclear magnetic resonances.

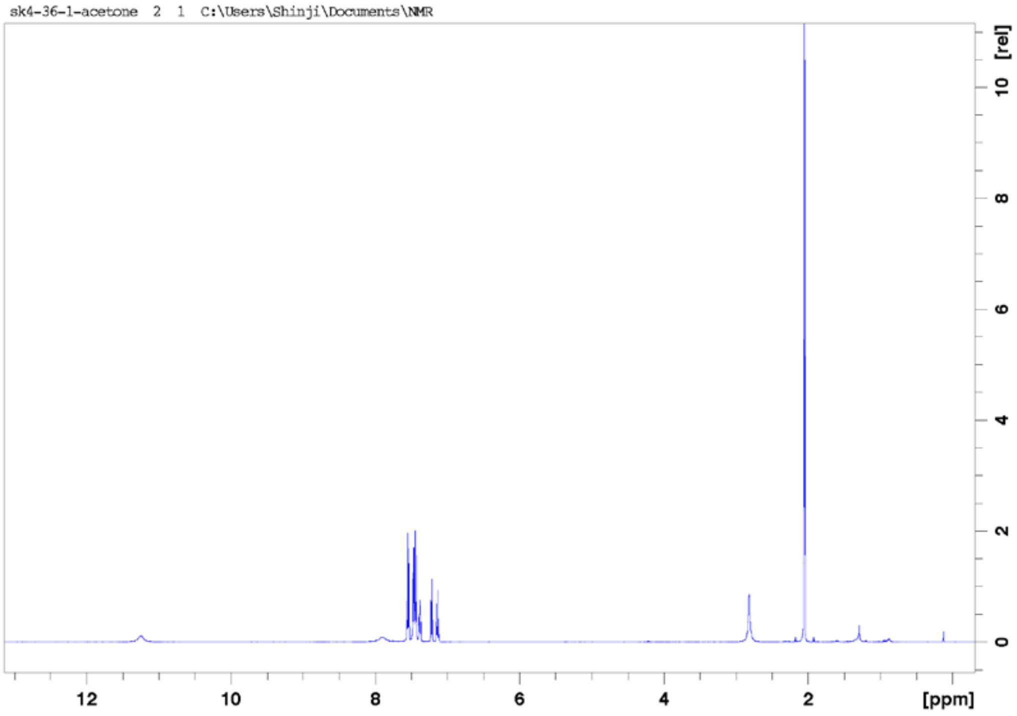

Supplementary Figure 17. ^{1}H NMR spectrum of **6** in acetone-d_6 (500 MHz).

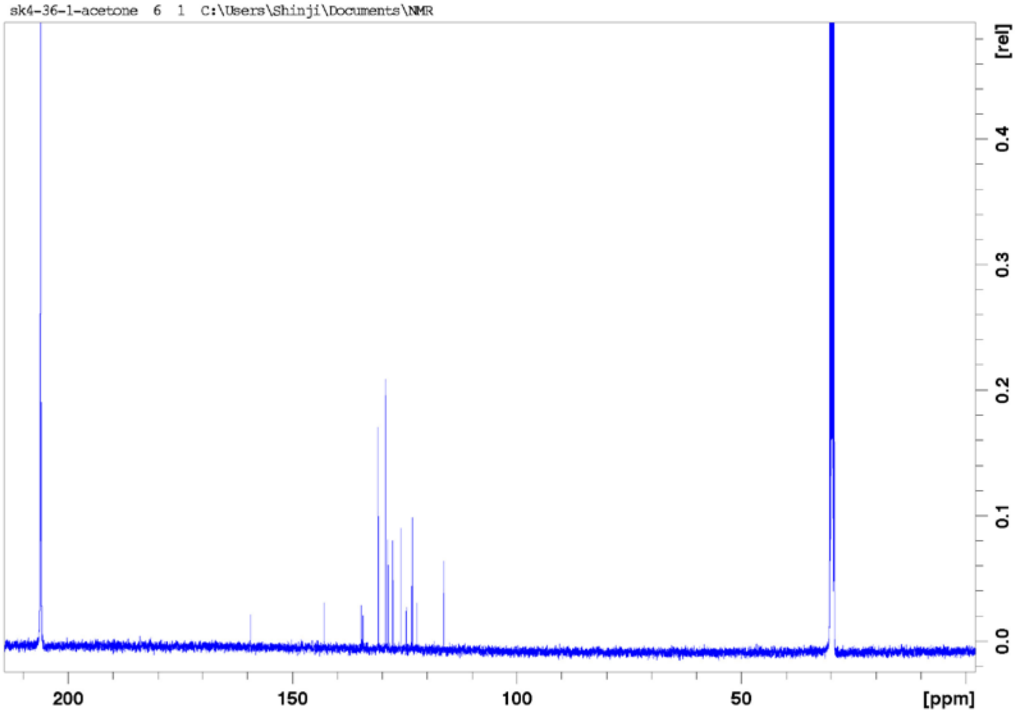

Supplementary Figure 18. ^{13}C NMR spectrum of **6** in acetone-d_6 (125 MHz).

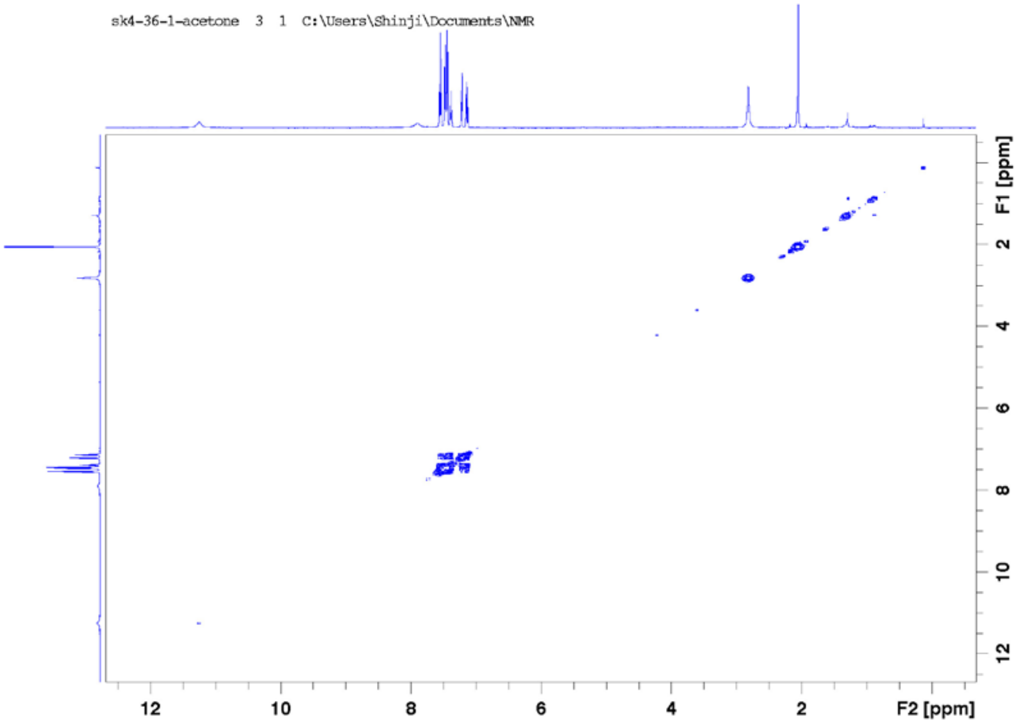

Supplementary Figure 19. COSY spectrum of **6** in acetone-d_6 (500 MHz). COSY, correlated spectroscopy.

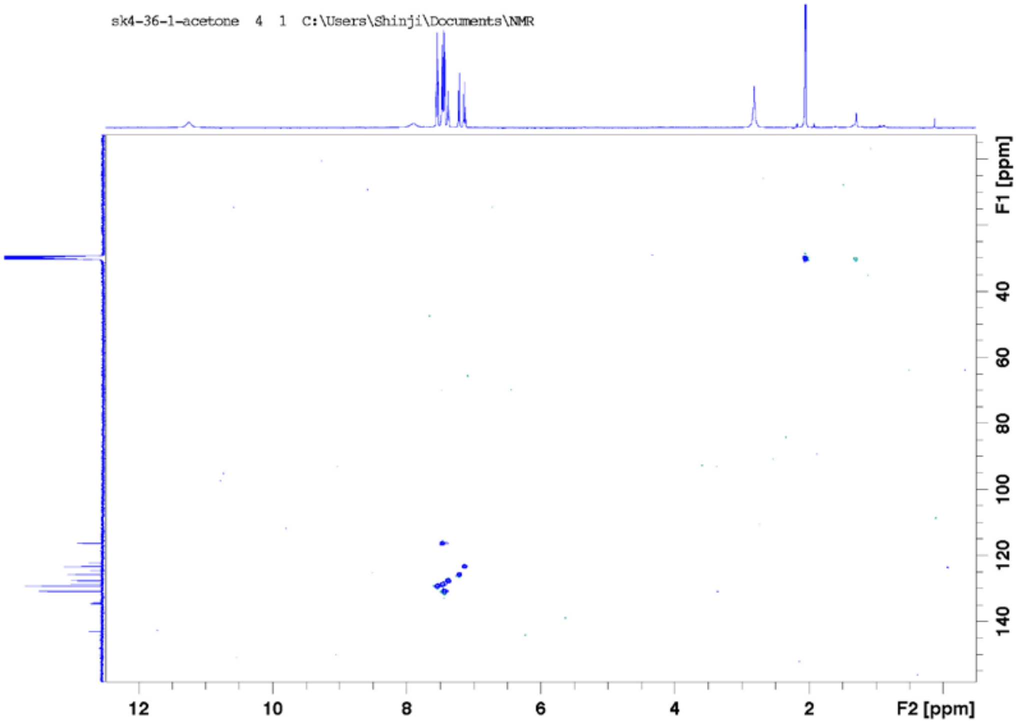

Supplementary Figure 20. HMQC spectrum of **6** in acetone-d_6 (500 MHz). HMQC, heteronuclear multiple quantum coherence.

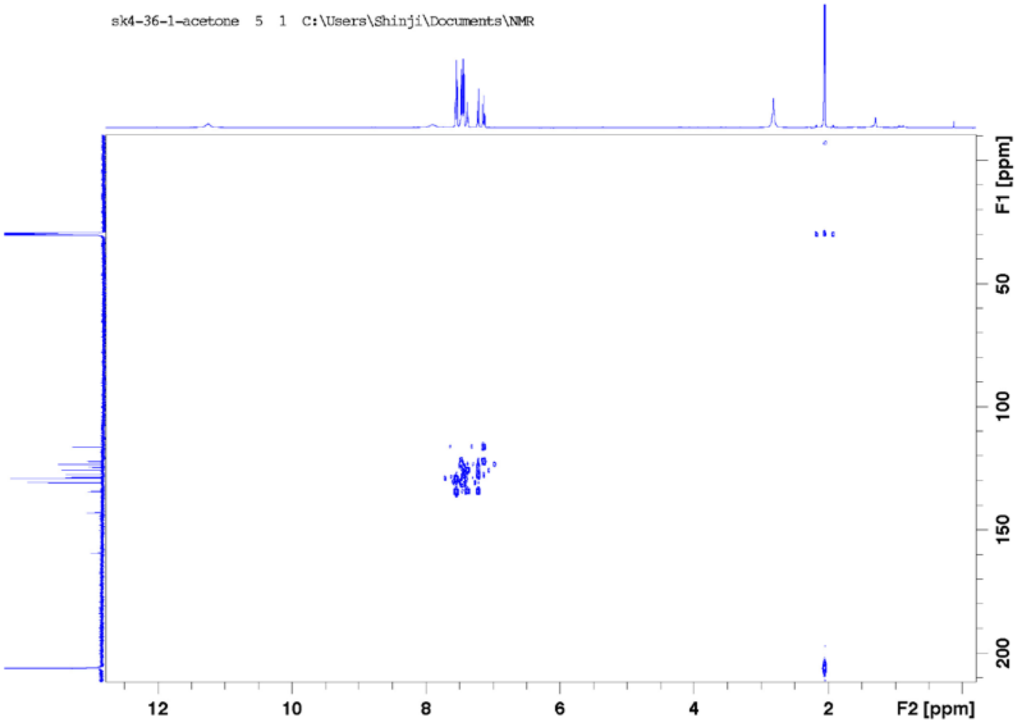

Supplementary Figure 21. HMBC spectrum of **6** in acetone-d_6 (500 MHz). HMBC, heteronuclear multiple bond correlation.

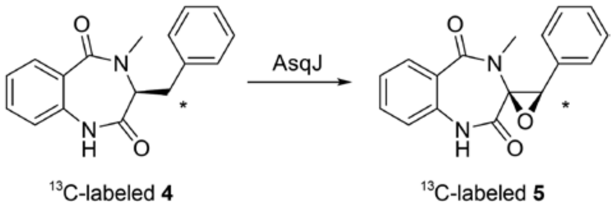

Supplementary Figure 22. Enzymatic preparation of ^{13}C-labeled cyclopenin **5** using AsqJ.

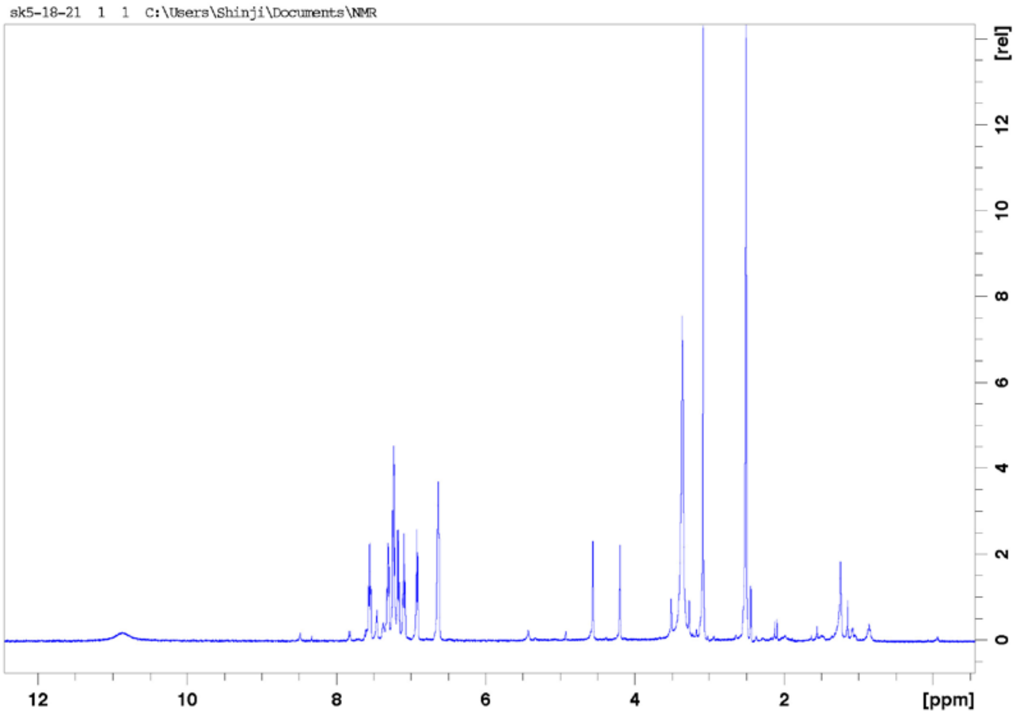

Supplementary Figure 23. ^{1}H NMR spectrum of ^{13}C-labeled **5** in DMSO-d_6 (500 MHz).

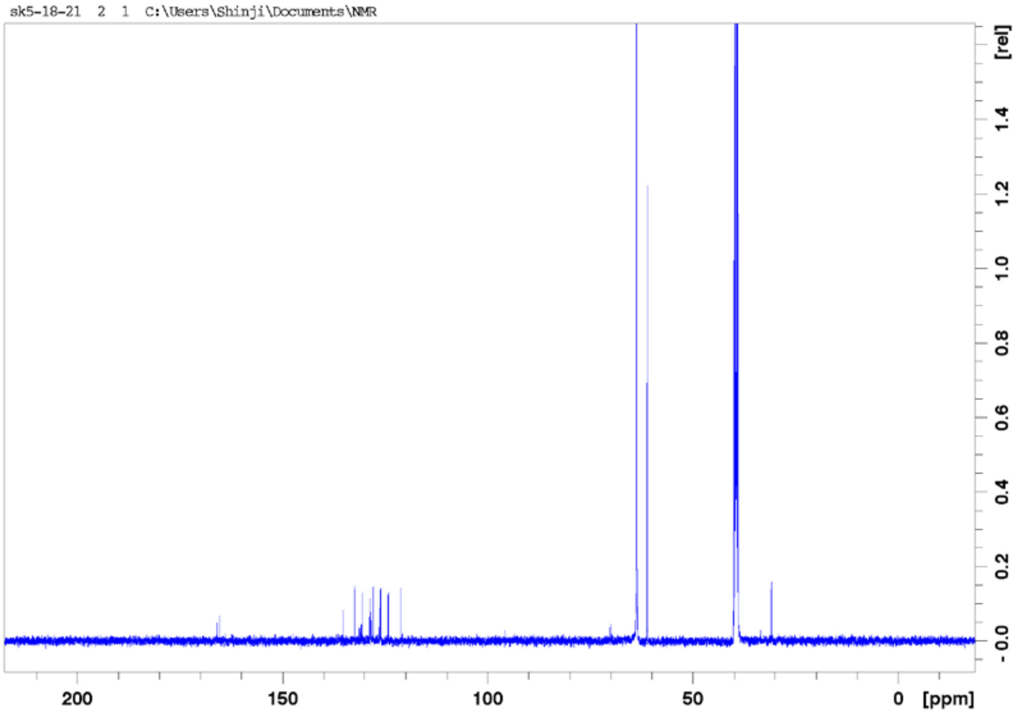

Supplementary Figure 24. ^{13}C NMR spectrum of ^{13}C-labeled **5** in DMSO-d_6 (125 MHz).

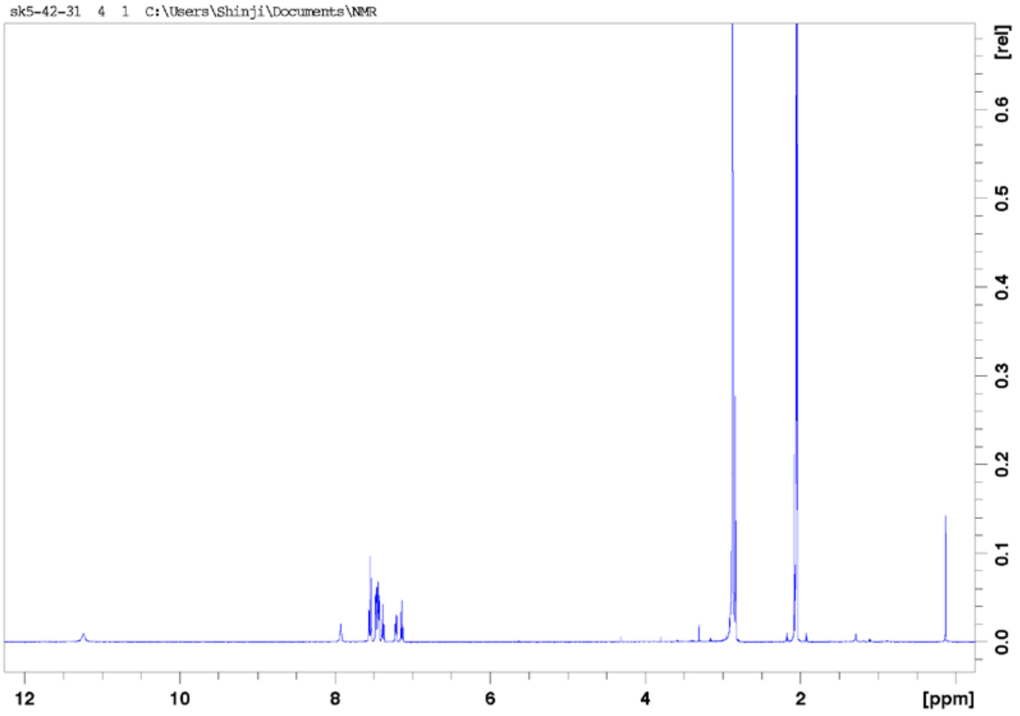

Supplementary Figure 25. ^{1}H NMR spectrum of ^{13}C-labeled **6** in acetone-d_6 (500 MHz).

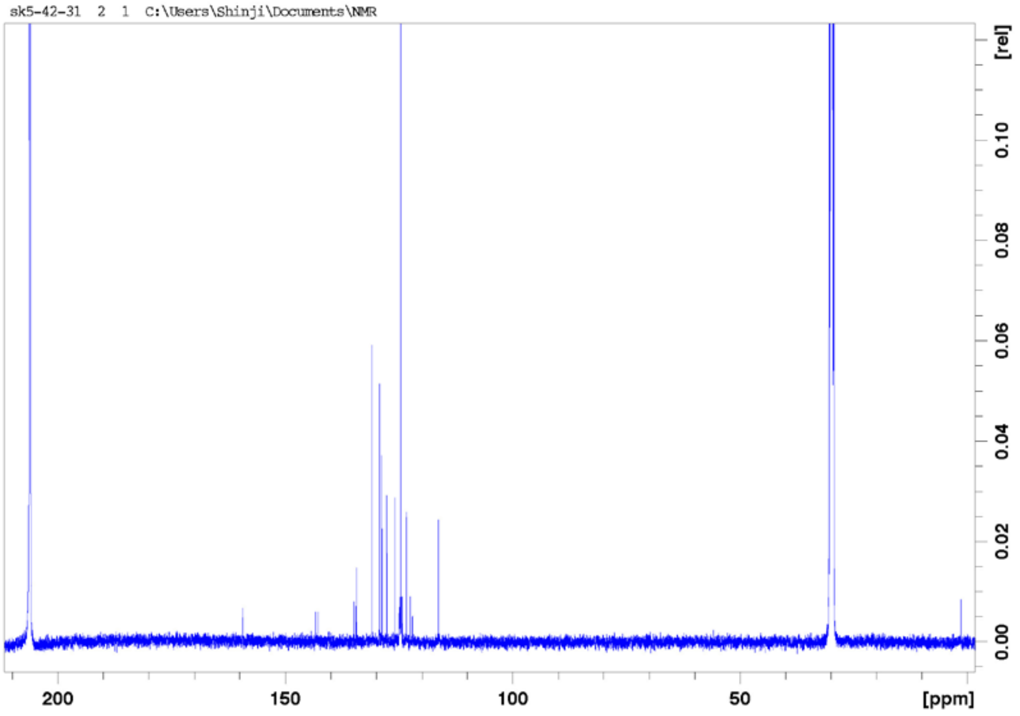

Supplementary Figure 26. ^{13}C NMR spectrum of ^{13}C-labeled **6** in acetone-d_6 (125 MHz).

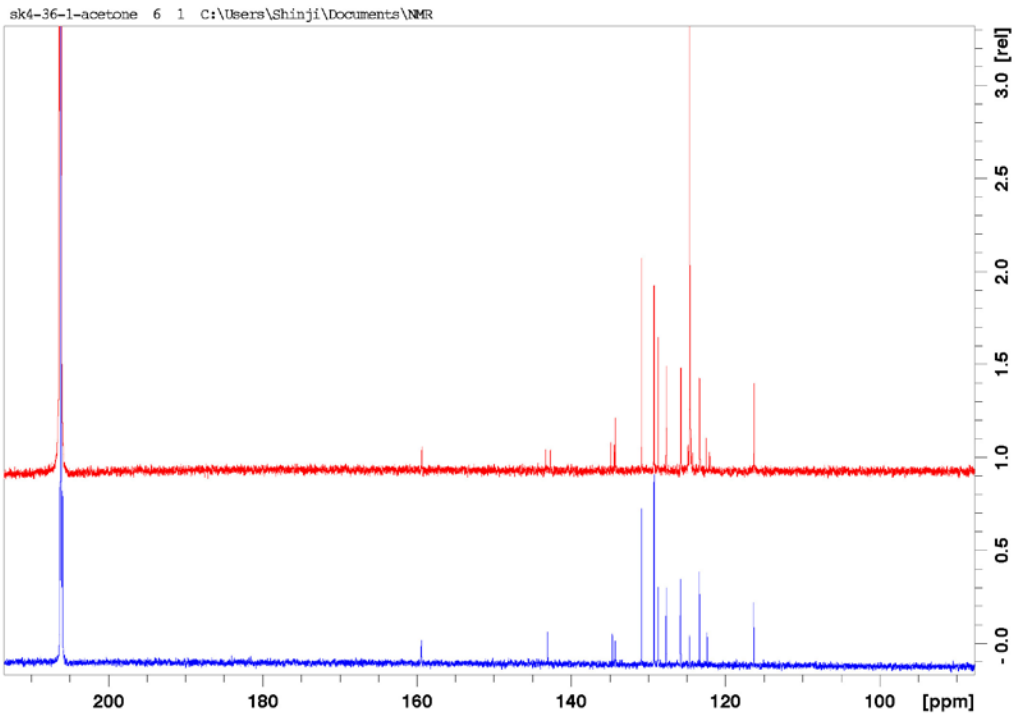

Supplementary Figure 27. Comparison of ^{13}C NMR spectrum of ^{13}C-labeled **6** and unlabeled **6** in acetone-d_6 (125 MHz). Top (red): the spectrum of ^{13}C-labeled **6**; bottom (blue): The spectrum of unlabeled **6**.

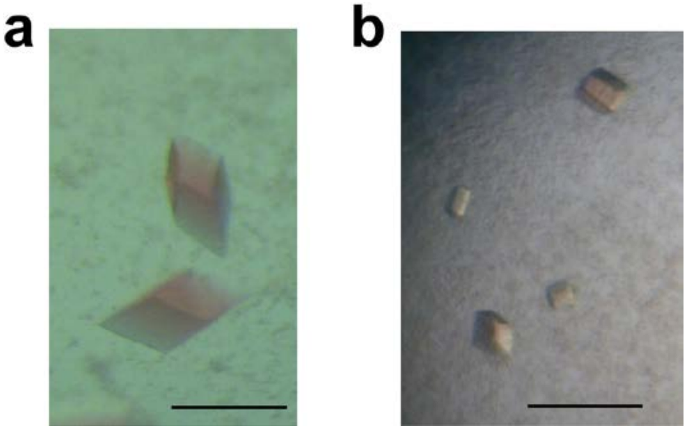

Supplementary Figure 28. Crystals of AsqI. (**a**) Crystals of apo AsqI. (**b**) Crystals of Zn^{2+}-bound AsqI. The scale bar shown in the image represents 0.1 mm.

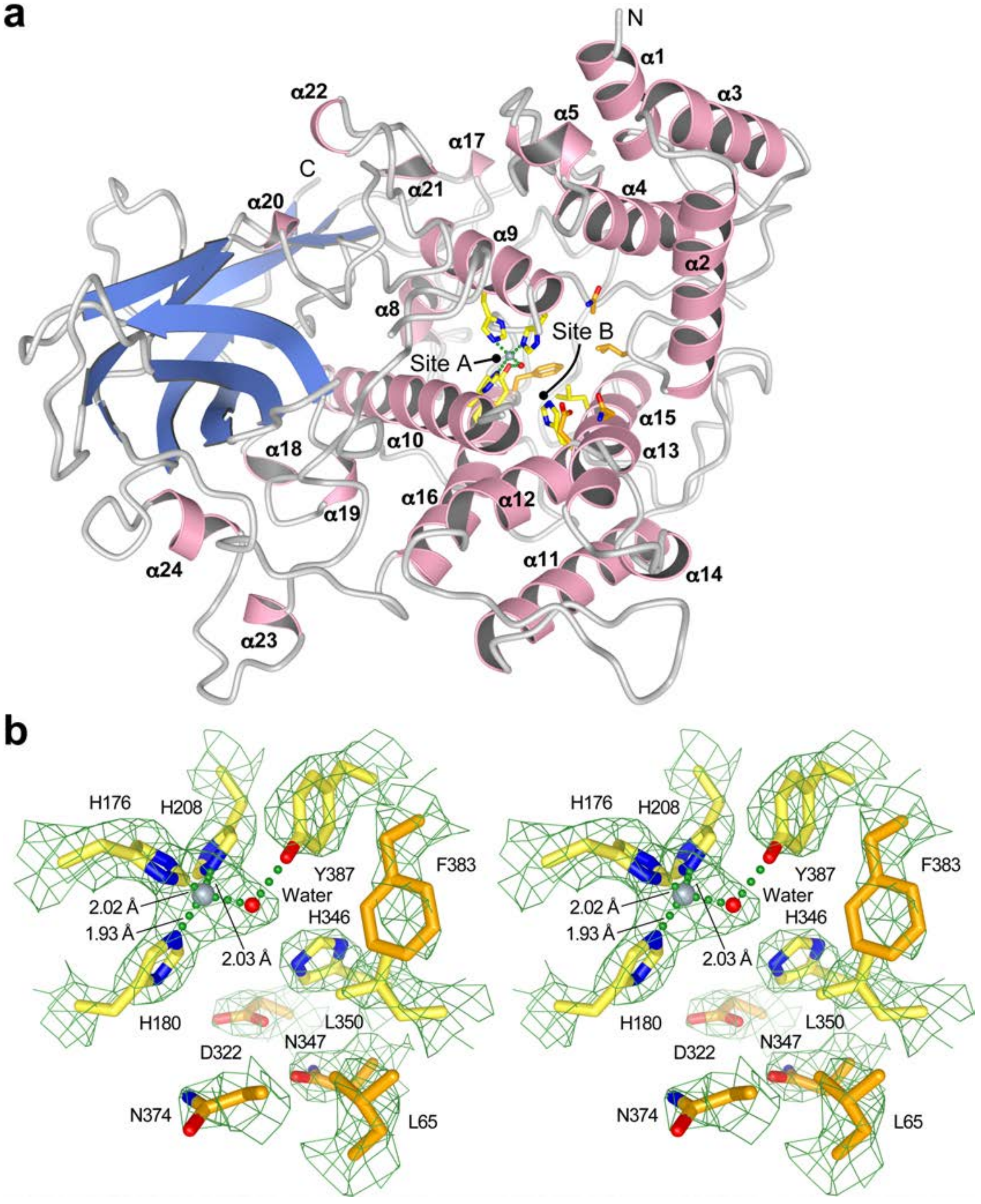

Supplementary Figure 29. The crystal structure of the AsqI–zinc complex. (**a**) The crystal structure of AsqI in complex with zinc ion. The overall fold of AsqI shows high resemblance to the hemocyanin fold. The coloring scheme is the same as in **Fig. 3a** except β-strands are colored in blue. (**b**) Stereo image of the electron density (2*F*o–*F*c map contoured at 1.5 σ, green mesh) of the side chains of the active-site residues, the zinc ion and the water molecule bound within the pocket.

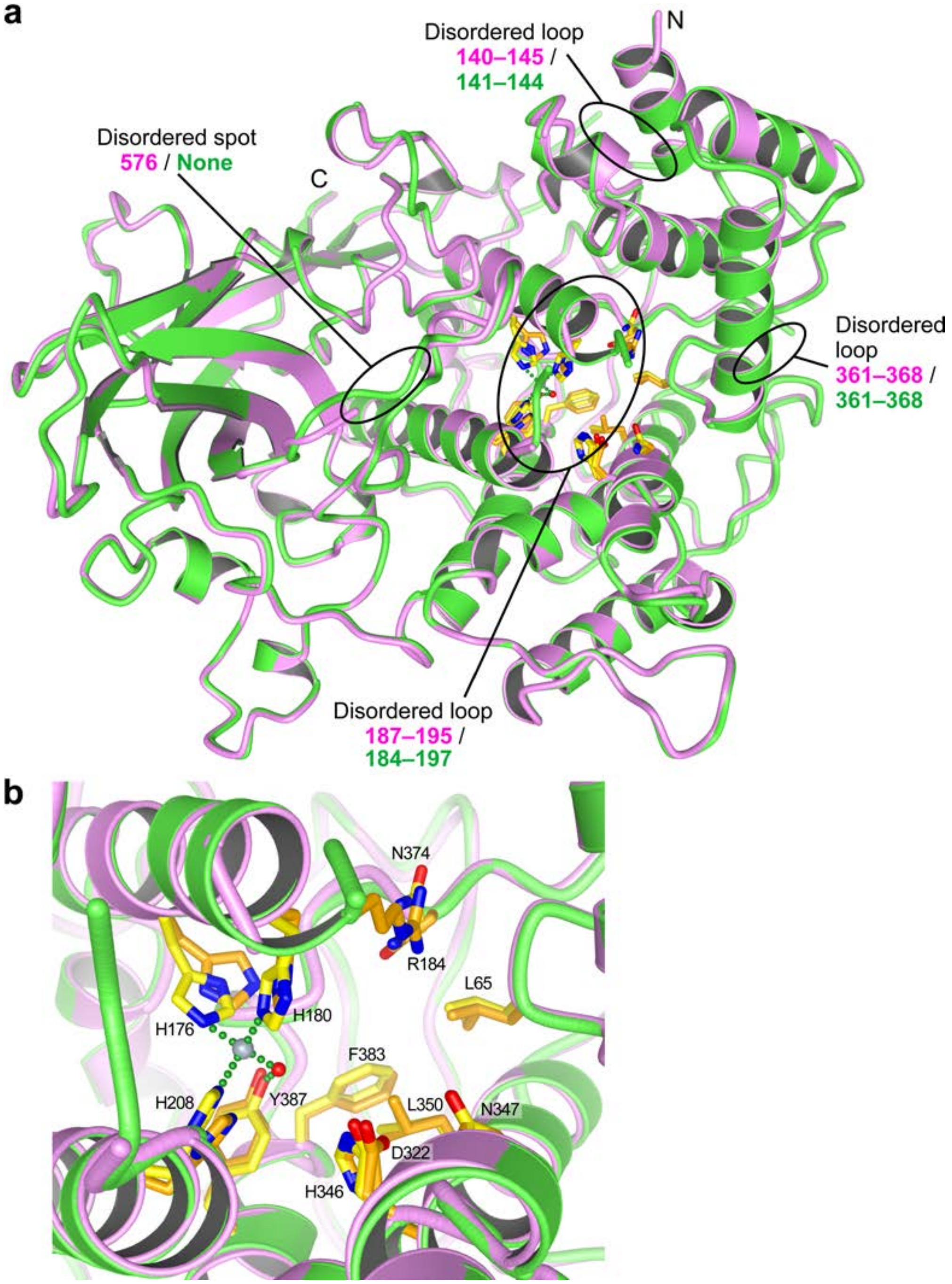

Supplementary Figure 30. Overlay of the crystal structures of the AsqI–zinc complex (magenta) and the apo AsqI (green). (**a**) The overall view and (**b**) the close-up of the zinc-binding site are given. Residue numbers of the disordered regions in the AsqI–zinc complex structure (magenta) and the apo AsqI structure (green) are given. The coloring scheme used to represent the side chains, ligands and their interactions is the same as in **Fig. 3a**.

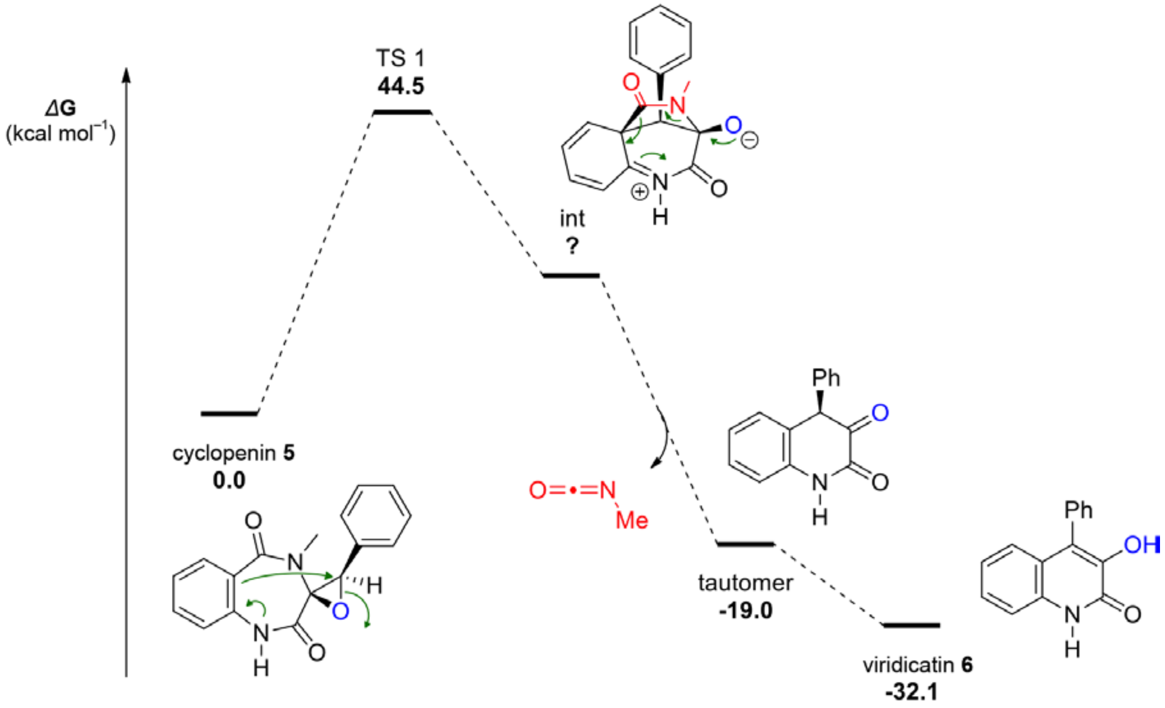

Supplementary Figure 31. Reaction pathway for the conversion of **5** to **6** in the absence of acid or base catalysts. Calculated free energy differences (⊿G) are shown in bold. Abbreviations are TS: transition state; Int: intermediate.

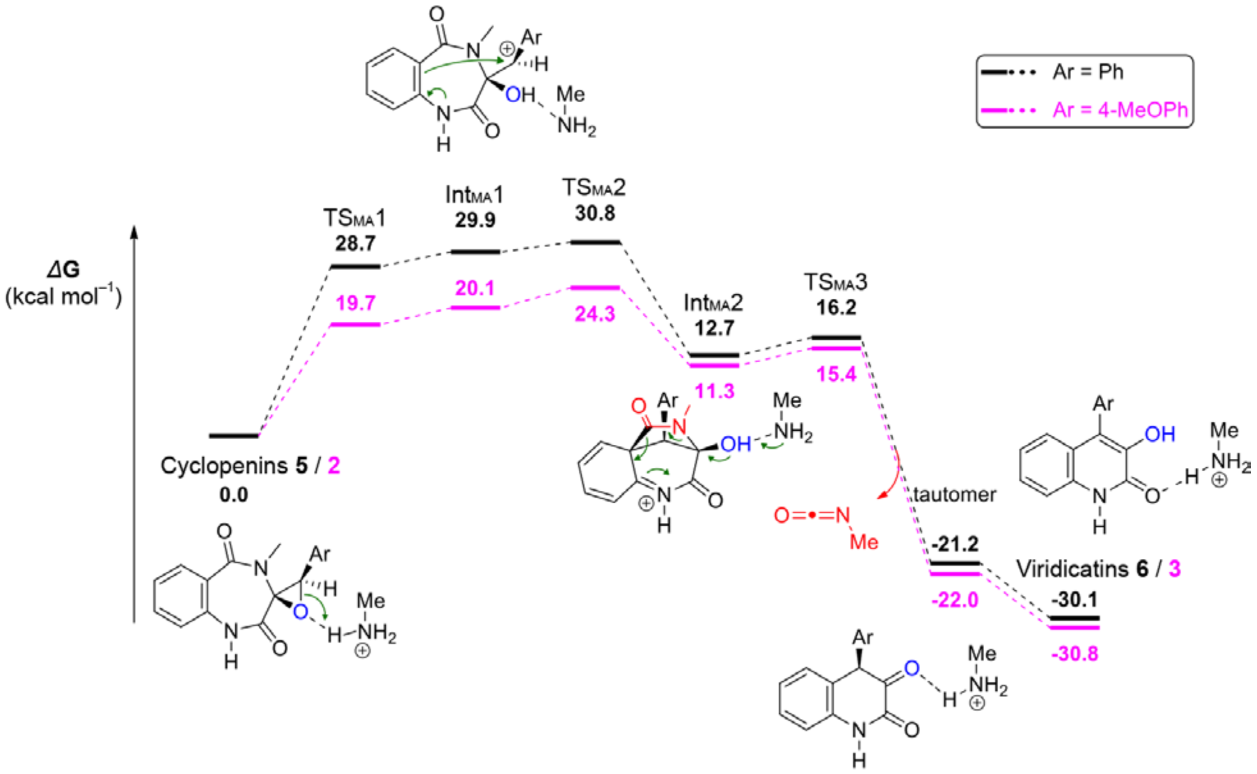

Supplementary Figure 32. Reaction pathway for the **5**-to-**6** (black) and **2**-to-**3** (pink) conversions in the presence of methylammonium, a model acid catalyst. Structures of ground-states and intermediates are shown. Abbreviations are $TA_{MA}1$: first transition state for methylammonium (MA)-catalyzed pathway; $TA_{MA}2$: second transition state for MA-catalyzed pathway; $TA_{MA}3$: third transition state for MA-catalyzed pathway; $Int_{MA}1$: intermediate 1 for MA-catalyzed pathway; $Int_{MA}2$: intermediate 2 for MA-catalyzed pathway.

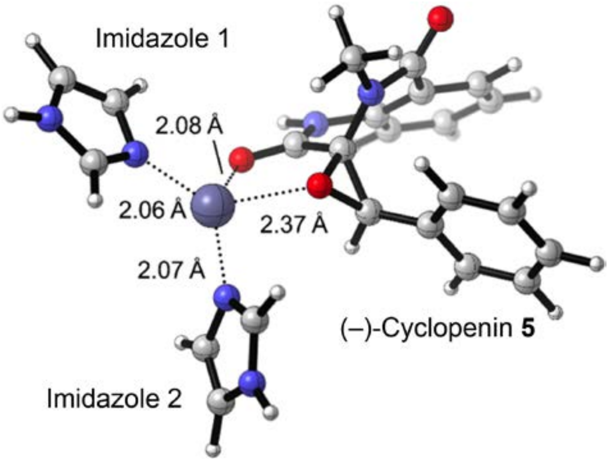

Supplementary Figure 33. The optimized structure of **5** in complex with two imidazole molecules and a tetrahedrally coordinated Zn^{2+}. The two imidazole molecules represent the two histidine side chains found in the predicted copper binding site of AsqI.

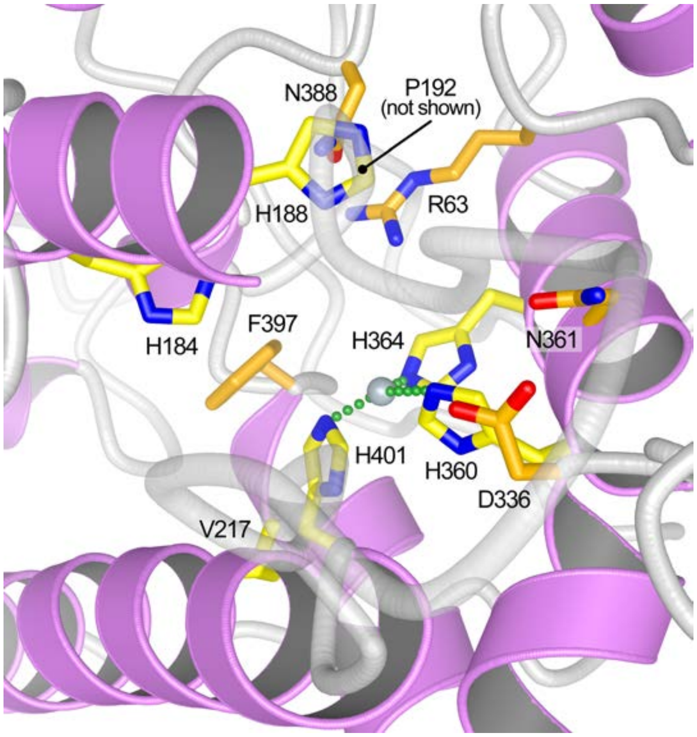

Supplementary Figure 34. A detailed view of the metal-binding site of the homology model of PngL. The bound metal is modeled in the metal-binding site B, which is fully equipped with three histidine residues (His360, His364, His401) that act as ligands for the bound metal. In contrast, the metal-binding site A in PngL is missing one of the three histidine residues (His184, His188, Val217). The loop spanning 191–209 is drawn in semi-transparent to clarify the view of the pocket. Residue 192, which corresponds to Arg184 in AsqI whose side chain forms a part of the ceiling of the active-site pocket, is a proline in PngL (not shown). Thus, the active site is less obstructed in this part of the pocket in PngL. The location and site of the active site pocket likely differ between PngL and AsqI due to those differences. The coloring scheme used to represent the α-helices, the side chains, ligands and their interactions is the same as in **Fig. 3a**.

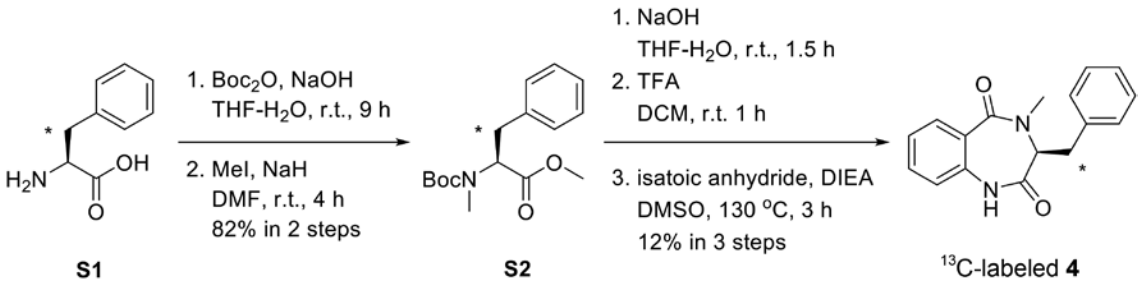

Supplementary Figure 35. Chemical synthesis of ^{13}C-labeled cyclopeptin **4**.

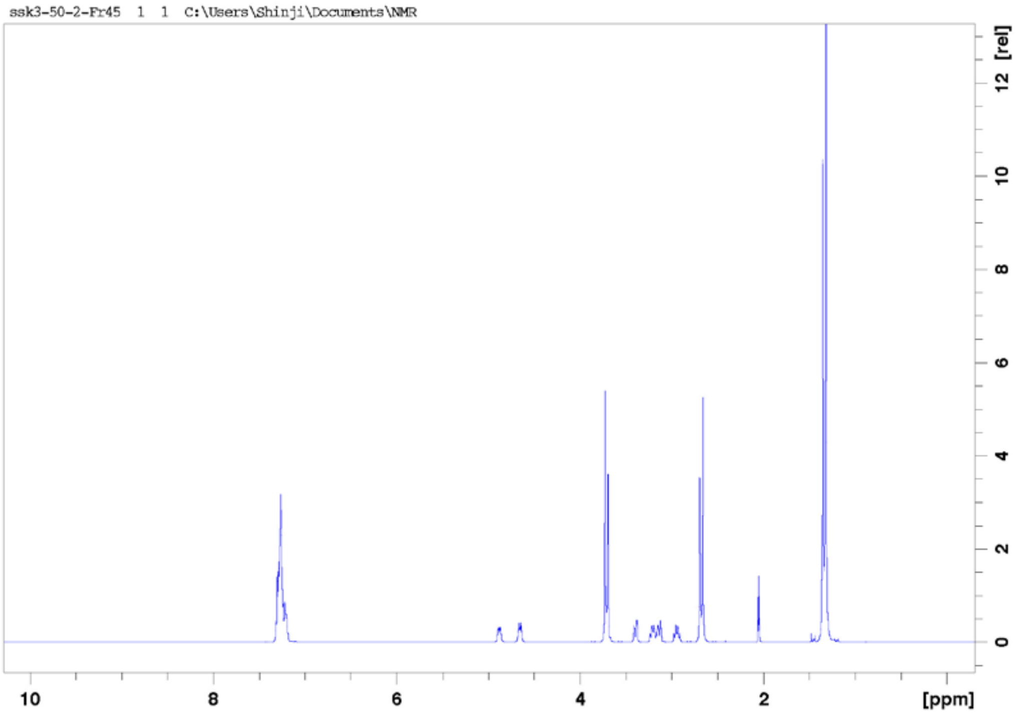

Supplementary Figure 36. ^{1}H NMR spectrum of **S2** in acetone-d_6 (500 MHz).

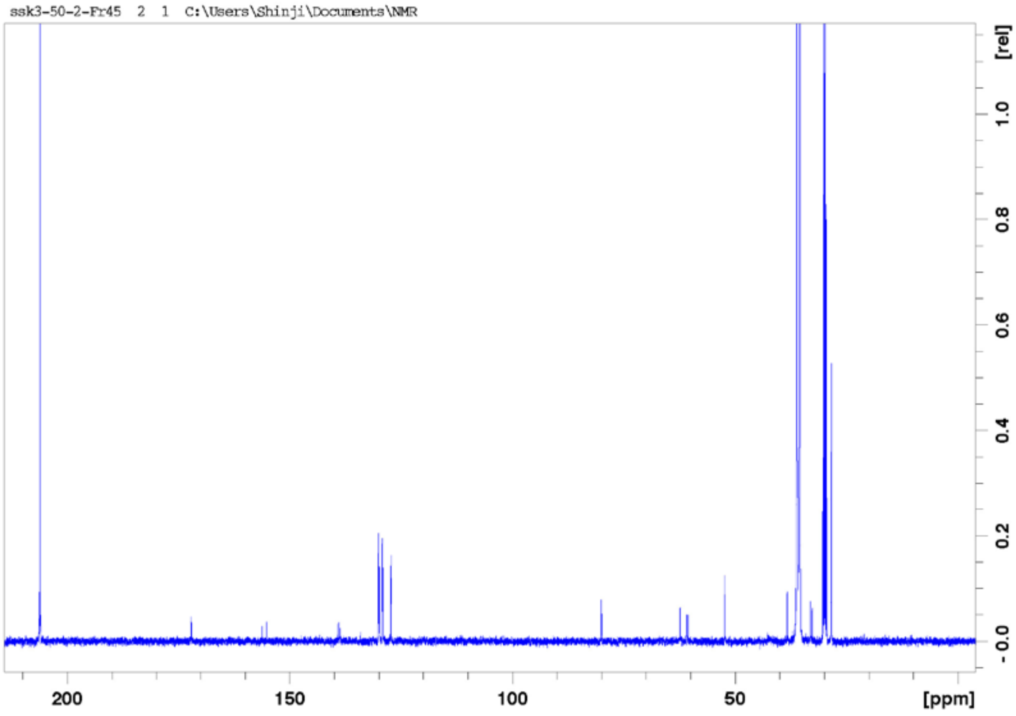

Supplementary Figure 37. ^{13}C NMR spectrum of **S2** in acetone-d_6 (125 MHz).

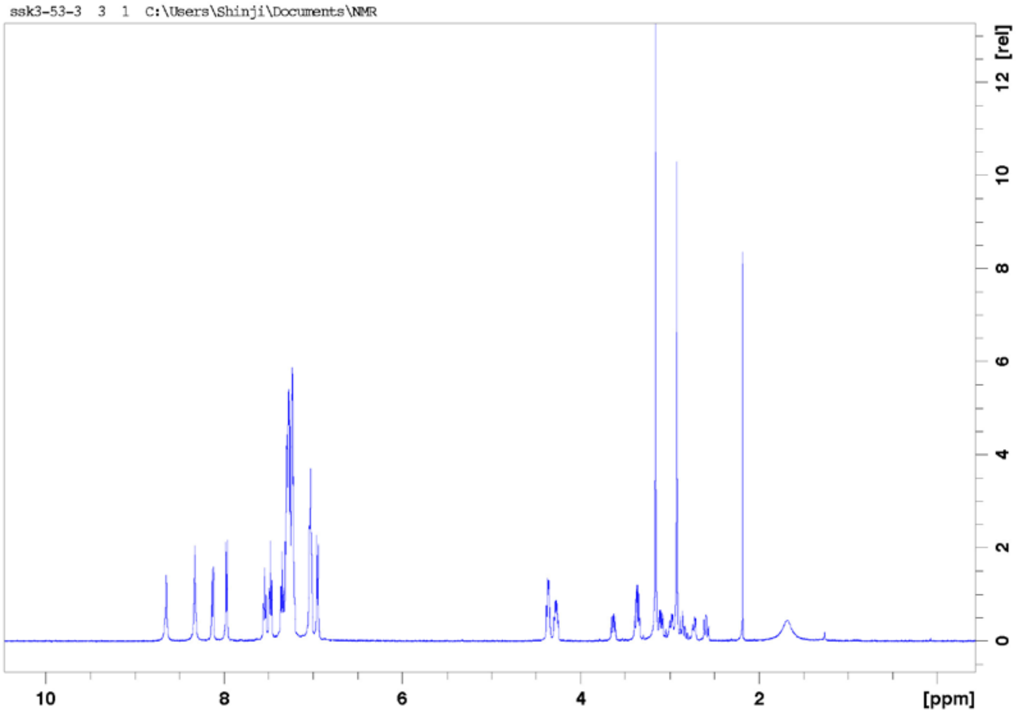

Supplementary Figure 38. ^{1}H NMR spectrum of ^{13}C-labeled **4** in $CDCl_3$ (500 MHz).

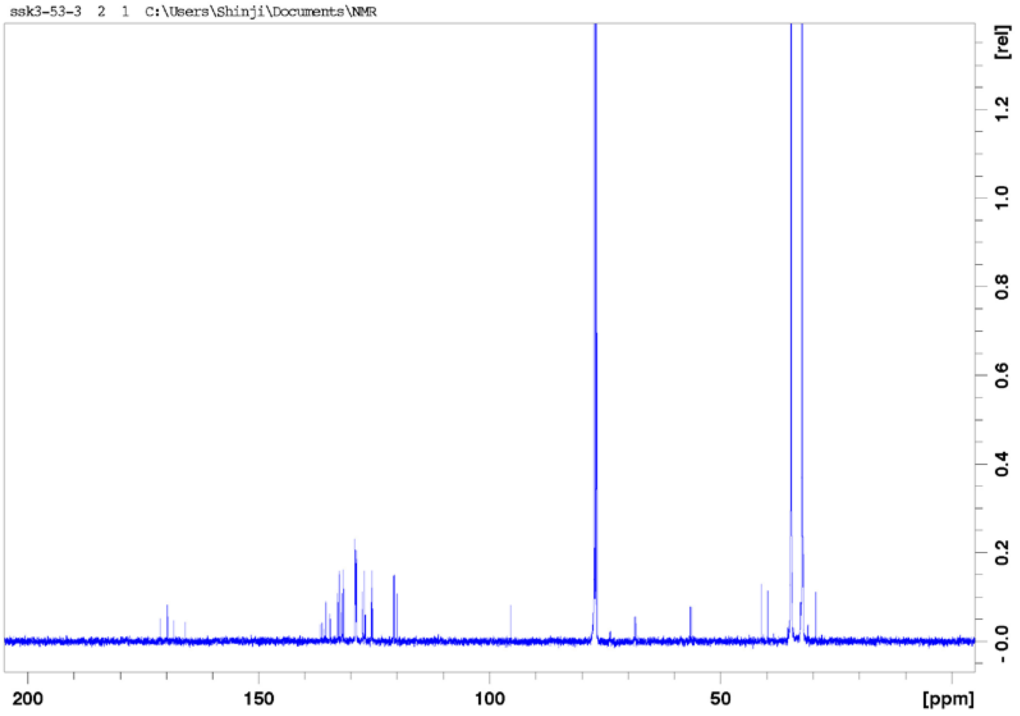

Supplementary Figure 39. ^{13}C NMR spectrum of ^{13}C-labeled **4** in $CDCl_3$ (125 MHz).

2. Supplementary Tables

Supplementary Table 1. Deduced functions of the *asq* and *png* genes.

Gene name	Protein name	Proposed protein function
asqA (AN9236)	AsqA	Transcription factor
asqB (AN9235)	AsqB	Hydrolase
asqC (AN9234)	AsqC	Terpene cyclase
asqD (AN9233)	AsqD	*O*-methyl transferase
asqE (AN9232)	AsqE	Short chain dehydrogenase
asqF (AN9231)	AsqF	FAD-dependent dehydrogenase
asqG (AN9230)	AsqG	FAD-dependent monooxygenase
asqP (AN11202)	AsqP	Prenyltransferase
asqH (AN11194)	AsqH	Prenyltransferase
asqO (AN11201)	AsqO	Hydrolase
asqI (AN11193)	AsqI	Hemocyanin
asqJ (AN9227)	AsqJ	α-KG dependent dioxygenase
asqK (AN9226)	AsqK	Nonribosomal peptide synthetase
asqL (AN9225)	AsqL	Cytochrome P450
asqM (AN9224)	AsqM	FAD/NADP-dependent monooxygenase
asqN (AN9223)	AsqN	*O*-methyl transferase

Gene name	Protein name	Proposed protein function
pngA	PngA	Prenyltransferase
pngB	PngB	FAD-dependent dehydrogenase
pngC	PngC	Prenyltransferase
pngD	PngD	Hydrolase
pngE	PngE	FAD-dependent monooxygenase
pngF	PngF	Short chain dehydrogenase
pngG	PngG	*O*-methyltransferase
pngH	PngH	Cytochrome P450
pngI	PngI	FAD/NADP-dependent monooxygenase
pngJ	PngJ	Nonribosomal peptide synthetase
pngK	PngK	α-KG dependent dioxygenase
pngL	PngL	Hemocyanin
pngM	PngM	*O*-methyltransferase

Supplementary Table 2. Oligonucleotide primer sequences. DNA primers were designed on the basis of sequence data obtained from the *A. nidulans* A1149 sequence database and draft genome of *Penicillium* sp. FKI-2140.

Primer name	Sequence, 5'–3'
5242F	CTTCATCCCCAGCATCATTACACCTCAGCAATGACTTGCACCCTTAGAGACCTCAATTC
5242R	CATACCCGTAATTTTCTGGGCATTTAAATAGAGATTTCCAATTGCAACTGCTGGCTG
pKW18244-Fw	CATCATCATCATCACATGACTTGCACCCTTAGAGACCTCAATTC
pKW18244-Rv	AGCAGCCGGATCCTAAGCCGGGTCCGTCCCTTTTGGACAC
5351FR	GTGATGATGATGATGATGGCTGCTG
pKW18227-pET28b	TAGGATCCGGCTGCTAACAAAGCCCG
pKW18239-pET28b	TAAGATCCGGCTGCTAACAAAGCCCG
pKW18255-Fw	CATCATCATCATCACATGTCCAACACTCCGGCGCGCTCTATC
pKW18255-Rv	AGCAGCCGGATCTTAGGCAGAATCGGTGCCTTTCGGACATG
AsqI-M4-F	GATTCGGCATATTATTGGCACATGGTATATCGCGGTGCTGGC
AsqI-M4-R	CAATAATATGCCGAATCATTCAAGTCGTAGTCGTCGCGCCAA
AsqI-M5-F	ATTGGGCAATGGTATATCGCGGTGCTGGCGGGGATAACTC
AsqI-M5-R	ATACCATTGCCCAATAATAATGCGAATCATTCAAG
AsqI-M6-F	GTACGTAGCTTCGCAGATGGTGGCTCGGTACGA
AsqI-M6-R	CTGCGAAGCTACGTACAAGAATACCTCCCCATG
AsqI-M7-F	GAACCTTGCTAACTATGGGCTCGGCAAGTTTGCTG
AsqI-M7-R	CATAGTTAGCAAGGTTCCCATATCGCTCTTCATCAA
AsqI-M14-F	GGTATATGCAGGTGCTGGCGGGGATAACTCCA
AsqI-M14-R	GCACCTGCATATACCATGTGCCAATAATAATGC
AsqI-M23-F	TCTCTTAGCCCAGTACCCCTCCTTGAACAAGCTGCT
AsqI-M23-R	TACTGGGCTAAGAGAATACCACCAACAAAGTTGAGA
AsqI-M24-F	CTTCACTTATATGGGCTCGGCAAGTTTGCTGAAAT
AsqI-M24-R	CCCATATAAGTGAAGGTTCCCATATCGCTCTTCAT
PngL-PM1-F	GATTTCGCATACTATTGGCATATGATGTTTCCGGG
PngL-PM1-R	ATAGTATGCGAAATCGTTGAAATCGTAATCTTCACGCC
PngL-PM2-F	TATTGGGCAATGATGTTTCCGGGAACGGCCGTGAA
PngL-PM2-R	CATCATTGCCCAATAGTAATGGAAATCGTTGAAATCG
PngL-PM3-F	GCAATTTGGCAAATTACGGTCACGGCAACTTCGCTG
PngL-PM3-R	CGTAATTTGCCAAATTGCCGTACAGATCGCCATCAAC
PngL-PM4-F	TACGGTGCAGGCAACTTCGCTGAGATTAGCTA
PngL-PM4-R	GTTGCCTGCACCGTAATTGTGCAAATTGCCGTA
PngL-PM5-F	CCCTGGGCAAAACATATCCAGTATTTTCGTCGTTT
PngL-PM5-R	ATGTTTTGCCCAGGGCCAGAAGCACGGGTC

Supplementary Table 3. NMR data of compound **9** in CD_3CN. The molecular formula of **9** was established by mass data [HRESIMS: *m/z* 168.0477 $(M+H)^+$, calcd. for $C_8H_{10}NOS^+$, 168.0478, Δ = 0.1 mmu].

Position	δ_H		mult. (*J* in Hz)	HMBC	δ_C
1					129.8
2	7.51	1H	m	4, 6	136.4
3	7.42	1H	m	1, 5	130.2
4	7.43	1H	m	2, 6	130.2
5	7.42	1H	m	1, 3	130.2
6	7.51	1H	m	2, 4	136.4
7					166.4
7-NH	6.25	1H	br		
8	2.72	3H	d (4.7)	7	28.1

Supplementary Table 4. NMR data of **6**[11] in acetone-d_6. The molecular formula of viridicatin and labeled viridicatin were established by mass data [HRESIMS: *m/z* 238.0863 $(M+H)^+$, calcd. for $C_{15}H_{12}NO_2$, 238.0860, Δ = 0.3 mmu, and *m/z* 239.0896 $(M+H)^+$, calcd. for $C_{15}H_{12}NO_2$, 239.0896 (^{13}C-containing), Δ = 0 mmu, respectively].

Position	δ_H		mult. (*J* in Hz)	HMBC	δ_C	J_{CC} observed in ^{13}C-labeled **6** (Hz)
1	11.25	1H	br s			
2					159.4	5.6
3					143.0	62.2
3-OH	7.90	1H	br s	4		
4					124.7	labeled
5	7.22	1H	dd (8.1, 1.2)	4, 7, 9	125.8	
6	7.14	1H	dd (7.6, 7.6)	8, 10	123.3	4.4
7	7.38	1H	ddd (7.6, 7.6, 1.2)	5, 9	127.7	
8	7.47	1H	m	6	116.3	2.7
9					134.3	
10					122.3	56.1
1’					134.7	54.7
2’	7.44	1H	m	4, 4’, 6’	130.9	
3’	7.55	1H	dd (7.2, 7.2)	1’, 5’	129.3	
4’	7.47	1H	m	2’, 6’	128.7	
5’	7.55	1H	dd (7.2, 7.2)	1’, 3’	129.3	3.6
6’	7.44	1H	m	4, 2’, 4’	130.9	

^{1}H and ^{13}C NMR spectra were recorded at 500 MHz and 125 MHz, respectively.

3. Supplementary Methods

Reagents, strains and general techniques for DNA manipulation. All chemicals were purchased from Sigma-Aldrich and Wako Pure Chemical Industries, Ltd. unless otherwise specified. Purchased chemicals were of reagent grade and used without further purification. *Penicillium* sp. FKI-2140 was provided by Prof. Satoshi Ōmura at Kitasato University. *Aspergillus nidulans* A1149 was obtained from the Fungal Genetics Stock Center, USA. *Escherichia coli* XL1-Blue (Agilent Technologies) and *Escherichia coli* TOP10 (Thermo Fisher Scientific) were used for plasmid propagation by standard procedures. Overproduction of recombinant proteins was carried out in *E. coli* BL21(DE3) (Thermo Fisher Scientific). DNA restriction enzymes were used as recommended by the manufacturer (Fermentas). The *A. nidulans* A1149 genomic DNA was prepared using the cetyltrimethylammonium bromide (CTAB) isolation buffer as described elsewhere[12]. PCR was carried out using PrimeSTAR GXL DNA polymerase (TAKARA Bio Inc.) as recommended by the manufacturers. Sequences of PCR products were confirmed through DNA sequencing (Macrogen Japan Corporation). Details of the plasmid construction are given below, and the plasmid maps are shown in **Supplementary Fig. 3**. *Saccharomyces cerevisiae* BY4741 was obtained from the Yeast Genetic Resource Center in Japan. *S. cerevisiae* BY4741 (genotype: *MAT*α *his3-Δ1 leu2-Δ0 met15-Δ0 ura3-Δ0*)[13] was used for homologous recombination-based molecular cloning of genes and plasmid assembly.

Construction of plasmids in this study. The open reading frame (ORF) of *asqI* and *pngL* were predicted based on the *A. nidulans* A1149 genome sequence information available from the National Center for Biotechnology Information (NCBI) database[14] and the draft genome sequence of *Penicillium* sp. FKI-2140 (see below for details), respectively. The predicted functions of the two ORFs were determined by comparison to known proteins using the BLAST peptide sequence database search program[15] and the Conserved Domain Database search[16]. We constructed two vectors for expressing *asqI* and *pngL*. The construction of those vectors was accomplished *in situ* using the endogenous homologous recombination activity of *S. cerevisiae*. For the construction of those vectors, pKW20093[17] carrying a *glaA* promoter[18] with the 2μ and the pBR322 origins of replication.

Construction of pKW5242 for isolation of the cDNA for AsqI. Initially, *asqI* and *asqO* were predicted to be encoded as a single gene in the original annotation of the *A. nidulans* genome sequence. Thus, the entire *asqI*- and *asqO*-coding regions, including the original terminator of *asqO* were amplified from the *A. nidulans* A1149 genomic DNA by PCR using the 5242F/5242R primer pair (**Supplementary Table 2**). The PCR product was combined with pKW20093[17], which was previously linearized by restriction digestion using *Sph* I (10 units), and introduced into *S. cerevisiae* BY4741 to form an intact plasmid *in situ* by homologous recombination. The resulting plasmid was amplified in *E. coli* for restriction digestion analysis. This plasmid was named pKW5242 (**Supplementary Fig. 3**) and the identity of the resulting vector was confirmed by DNA sequencing (Macrogen Japan Corporation). This plasmid was used for preparation of the cDNA for the full-length coding region of *asqI*.

Construction of pKW18244 for expression of *asqI*. *A. nidulans* A1149 harboring pKW5242 was grown in CD-ST liquid medium containing pyridoxine hydrochloride (0.5 µg mL^{-1}) and riboflavin (1.25 µg mL^{-1}) for 2 days, and total RNA was isolated from the mycelia using the Ambion RNAqueous® kit (Thermo Fisher Scientific Inc.). The SMARTer RACE cDNA Amplification kit (Clontech Laboratories, Inc.) was used for synthesizing a cDNA library from the isolated total RNA according to the protocol supplied by the manufacturer. The cDNA of *asqI* was amplified from the cDNA library by PCR using the primer pair pKW18244-Fw/pKW18244-Rv (**Supplementary Table 2**). Separately, linearized pET28b(+) expression vector (EMD Millipore Corporation) was amplified using the 5351FR/pKW18227-pET28b primer set (**Supplementary Table 2**). These two amplicons were combined to generate pKW18244 (**Supplementary Fig. 3**) using the GeneArt Seamless Cloning and Assembly kit (Thermo Fisher Scientific). The identity of the resulting vector was confirmed by DNA sequencing (Macrogen Japan Corporation). This plasmid was used for expression of *asqI* in *E. coli* for the preparation of recombinant AsqI for *in vitro* assays.

Construction of pKW18255 for expression of *pngL*. Isolation of genomic DNA from *Penicillium* sp. FKI-2140 was performed using Genomic-tip kit (QIAGEN) as recommended by the manufacture. Purified genomic DNA was sequenced on Miseq instrument (Illumina) by the DNA sequencing facility at Toyohashi University of Technology. Sequenced data was annotated

with Migap (http://www.migap.org/) using the *A. oryzae* genome sequence database[19] as a reference, and analyzed with i*n silico* Molecular Cloning software (in silico biology, inc.). The draft genome sequence was used to predict the sequence of *pngL*. The *pngL* gene whose codon was optimized for its expression in *E. coli* was synthesized by eurofins Genomics. The synthesized *pngL* was amplified by PCR using two primers pKW18255-Fw/pKW18255-Rv (**Supplementary Table 2**). The pET28b(+) expression vector (EMD Millipore Corporation) was also amplified using the 5351FR/pKW18239-pET28b primer set. These amplicons were combined to generate pKW18255 (**Supplementary Fig. 3**) using GeneArt Seamless Cloning and Assembly kit (Thermo Fisher Scientific). The identity of the resulting vector was confirmed by DNA sequencing (Macrogen Japan Corporation). This plasmid was used for expression of *pngL* in *E. coli* for the preparation of recombinant PngL for *in vitro* assays.

Construction of plasmids for expression of AsqI mutants. The oligonucleotide primers used for generating the mutants are listed in **Supplementary Table 2**. The plasmid pKW18244 containing the wild-type *asqI* gene was used as the template for PCR-based site-directed mutagenesis. Two primers AsqI-M4-F and AsqI-M4-R were for His176Ala mutation, and the resulting plasmid carrying the mutant gene was named pKW18244-M4. His180Ala and His208Ala mutants of AsqI were constructed in the same manner using the primers pairs AsqI-M5-F/AsqI-M5-R and AsqI-M6-F/AsqI-M6-R, respectively. His346Ala, Arg184Ala, Asp322Lue and Asn347Lue mutants of AsqI were constructed in the same manner using the primers pairs AsqI-M7-F/AsqI-M7-R, AsqI-M14-F/AsqI-M14-R, AsqI-M23-F/AsqI-M23-R and AsqI-M24-F/AsqI-M24-R, respectively. The accuracy of the DNA sequences of the coding regions of the plasmids prepared here, including the mutagenized positions, was confirmed by DNA sequencing (Macrogen Japan Corporation).

Construction of plasmids for expression of PngL mutants. The oligonucleotide primers used for generating the mutants are listed in **Supplementary Table 2**. The plasmid pKW18255 containing the wild-type *pngL* gene was used as the template for PCR-based site-directed mutagenesis. Two primers PngL-PM1-F and PngL-PM1-R were for His184Ala mutation, and the resulting plasmid carrying the mutant gene was named pKW18255-PM1. His188Ala, His360Ala, His364Ala and His401Ala mutants of PngL were constructed in the same manner using the

primers pairs PngL-PM2-F/PngL-PM2-R, PngL-PM3-F/PngL-PM3-R, PngL-PM4-F/PngL-PM4-R and PngL-PM5-F/PngL-PM5-R, respectively. The accuracy of the DNA sequences of the coding regions of the plasmids prepared here, including the mutagenized positions, was confirmed by DNA sequencing (Macrogen Japan Corporation).

Expression and purification of AsqI, PngL and their mutants. Overexpression and subsequent protein purification of AsqI was performed as follows: BL21(DE3) harboring plasmid pKW18244 was grown overnight in 20 mL of LB medium with 50 µg mL^{-1} kanamycin at 37 °C. Five liters of fresh LB medium with 50 µg mL^{-1} kanamycin was inoculated with 20 mL of the overnight culture and incubated at 37 °C until the optical density at 600 nm (OD_{600}) reached 0.6. Then expression of the gene was induced with 100 µM isopropylthio-β-D-galactoside (IPTG) at 15 °C. Incubation was continued for another 24 h, after which cells were harvested by centrifugation at 10,000 × *g* for 5 min. All subsequent procedures were performed at 4 °C or on ice. Harvested cells were resuspended in disruption buffer (0.1 M Tris-HCl at pH 7.4, 0.1 M NaCl and 20 mM imidazole). Cells were disrupted by French Press, and the lysate was clarified by centrifugation at 10,000 × *g* for 10 min. The supernatant and precipitate were recovered as the soluble and insoluble fraction, respectively. The soluble fraction containing protein was applied onto a Ni Sepharose 6 Fast Flow (GE Healthcare) column. After washing the column with 30 mM imidazole and 0.1 M NaCl in 0.1 M Tris-HCl pH 7.4, the target protein was eluted with 0.06 to 1.0 M imidazole and 0.1 M NaCl in 0.1 M Tris-HCl pH 7.4. Fractions with the desired protein, which typically eluted at 0.2 M imidazole, were pooled and further concentrated with Amicon Ultra centrifugal concentrator (EMD Millipore Corporation). Then, the protein was further purified by Superdex 200 gel filtration (16 × 600 mm, GE Healthcare) in a buffer containing 10 mM Tris-HCl, pH 7.4 and 0.1 M NaCl at a flow rate of 1 mL min^{-1}. The purified proteins in 10 mM Tris-HCl (pH 7.4) and 0.1 M NaCl were pooled and concentrated to a concentration of 25 mg mL^{-1} with Amicon Ultra centrifugal concentrator. Protein concentration was estimated using the Bio-Rad protein assay kit with bovine serum albumin as a standard. This sample was analyzed by sodium dodecyl sulfate (SDS)–polyacrylamide gel electrophoresis (PAGE) using Tris-HCl 10% of polyacrylamide gel stained with Coomassie Brilliant Blue R-250 stain solution (CBB Stain One Super; nacalai tesque) (**Supplementary Fig. 4**, lane 1). The AsqI mutants and the wild-type PngL were overexpressed using essentially the same procedure as

described above. However, there was a difficulty in purifying those proteins (**Supplementary Fig. 4**, lanes 2–9). Therefore, only partially purified samples of those proteins were used for further studies. All of the PngL mutants went into the insoluble fraction and could not be isolated as stable soluble proteins for detailed kinetic analyses (**Supplementary Fig. 4**, lanes 10–19).

Kinetic analysis of PngL. Different concentrations of **2** (0.05, 0.1, 0.2, 0.4 and 0.8 mM) was mixed with partially purified PngL in MES-Na buffer in a total reaction volume of 40 µL. After 2 min. of incubation at 30 °C, the reaction was quenched with 80 µL of EtOAc containing 10 µM of anthraquinone as an IS. The organic layer was separated by centrifugation, and the isolated organic fraction was dried *in vacuo*. The dried material was subjected to LC–MS analysis as described earlier. Initial reaction rates were determined on the basis of the amount of **3** present in the sample, and the data points were plotted as shown in **Supplementary Fig. 8**. Kinetic parameters were calculated by nonlinear regression of the data using GraphPad Prism software (GraphPad Software, Inc.). Each data point is a mean of triplicate measurements. The standard deviation is given in the plot as an error bar at each data point.

Protein crystallization and data collection. A broad screening of crystallization conditions was performed for AsqI using a sitting drop method. Crystallization conditions that yielded microcrystals were reproduced and optimized in terms of pH, precipitant concentration and drop volume. The best crystal of apo AsqI was obtained by mixing 1.0 µL of the protein (40 µM) with 1.0 µL of a mother liquor of Morpheus® 2-46: 0.02 M DL-Glu, 0.02 M DL-Ala, 0.02 M DL-Ser, 0.02 M DL-Lys HCl, 0.02 M Gly, 12% (v v^{-1}) ethylene glycol, 6% (w v^{-1}) PEG 8000 and 100 mM Tris/Bicine at pH 8.5. Crystals appeared after a few days at 20 °C and grew as octahedral crystals (**Supplementary Fig. 28a**). On the other hand, the best crystal of zinc-bound AsqI was obtained by mixing 1.0 µL of the protein (40 µM) with 1.0 µL of a mother liquor of Wizard Precipitant Synergy-tubes 132: 6.6% (v v^{-1}) PEG 400, 9.9% (w v^{-1}) PEG 1500 and 0.1 M HEPES/ NaOH at pH 7.5. Crystals of the Se-Met derivative were obtained in the same condition as Zn^{2+}-bound AsqI except for the concentration of PEG 400 (4.65% (v v^{-1})) and PEG 1500 (6.6% (v v^{-1})). Crystals appeared after a few days at 20 °C and grew as octahedral crystals (**Supplementary Fig. 28b**). Prior to data collection, crystals were transferred to a cryoprotectant solution (14% ethylene glycol and 16% PEG 400 in the mother liquor for apo AsqI and zinc-

bound AsqI, respectively) and immediately cryo-cooled in liquid nitrogen. All data were collected at the temperature of 95 K under the stream of low-temperature nitrogen gas at the beamline BL-17A of the Photon Factory, Tsukuba, Japan. The X-ray wavelength used to collect diffraction from the native apo and zinc-complexed AsqI crystals was 0.98000 Å, while that used to collect diffraction from crystals of the Se-Met derivative was 0.97911 Å. Crystals of AsqI belonged to the space group *I*222 and had one molecule per asymmetric unit. X-ray diffraction data were processed with the programs XDS[20] and AIMLESS[21].

Construction of the homology model of PngL. The homology model of another cyclopenase-type enzyme PngL (**Supplementary Fig. 34**) was constructed from the crystal structures of AsqI and the *Manduca sexta* (tobacco hornworm) prophenoloxidase[4] using MODELLER[22]. The structural refinement of the homology model was performed with ModRefiner[23].

General methods and procedures of chemical synthesis. All reactions involving air or moisture sensitive reactants were conducted under a positive pressure of dry nitrogen. All commercially available solvents, chemicals, biochemicals, and reagents were reagent grade and used as supplied unless otherwise stated. Removal of solvent was performed under reduced pressure, at below 30 °C, using an EYELA rotary evaporator. All reactions and fractions from column chromatography were monitored by thin layer chromatography (TLC). Analytical TLC was done on glass plates (5 × 1.5 cm) pre-coated (0.25 mm) with silica gel (normal SiO_2, Merck 60 F254). Compounds were visualized by exposure to UV light. Flash chromatography was performed on silica gel (Cica, Silica Gel 60 N, 40–50 μm).

NMR spectra were obtained with a Bruker BioSpin AVANCE III HD 500 MHz spectrometer (^{1}H 500 MHz, ^{13}C 125 MHz). ^{1}H NMR chemical shifts are reported in parts per million (ppm) using the proton resonance of residual solvent as references: $CDCl_3$ δ 7.26, acetone-d_6 δ 2.05, DMSO-d_6 δ 2.50 and CD_3CN δ 1.94[24]. ^{13}C NMR chemical shifts are reported relative to $CDCl_3$ δ 77.16, acetone-d_6 δ 29.84, DMSO-d_6 δ 39.52 and CD_3CN δ 1.32[24]. Mass spectra were recorded with a Thermo SCIENTIFIC ACCELA Exactive liquid chromatography–mass spectrometer (LC–MS) by using both positive and negative electrospray ionization (ESI). Samples were separated for analysis on an ACQUITY UPLC 1.8 μm, 2.1 × 50 mm C18 reversed-phase column (Waters)

using a linear gradient of 5–100% (v v^{-1}) MeCN in H_2O supplemented with 0.05% (v v^{-1}) formic acid at a flow rate of 0.5 mL min^{-1}. Optical rotations were measured on a JASCO P-2200 digital polarimeter.

Chemical synthesis of ^{13}C-labeled cyclopeptin 4. The scheme for the synthesis of ^{13}C-labeled **4** from C3-labeled L-phenylalanine **S1** is shown in **Supplementary Fig. 35**. To a stirred solution of **S1** (69.6 mg, 0.419 mmol) in 1 M aq. NaOH (1.05 mL) was added Boc_2O (98.7 mg, 0.452 mmol) in THF (1 mL). After being stirred for 9 h at room temperature, the reaction was quenched with 0.5 M aq. sodium dihydrogen citrate (2.5 mL) and brine (2 mL) and was extracted with EtOAc (2 × 10 mL). Combined organic layers were washed with brine (10 mL), dried over Na_2SO_4 and concentrated *in vacuo* to yield crude product (114.4 mg). This was dissolved in dry DMF (4.3 mL) and then 50% sodium hydride in oil (49.8 mg, 1.04 mmol) and methyl iodide (100 µL, 1.6 mmol) were added to the solution. After being stirred for 4 h at room temperature, the reaction was quenched with 0.5 M aq. sodium dihydrogen citrate (4 mL) and was extracted with EtOAc (2 × 10 mL). Combined organic layers were washed with brine (2 × 6 mL), dried over Na_2SO_4 and concentrated *in vacuo*. The residue was purified by SiO_2 column chromatography (*n*-hexane:acetone = 10:1) to yield **S2** (101.0 mg, 0.343 mmol, 81.9%); **S2** exists as two conformers in acetone-d_6. ^{1}H NMR (acetone-d_6, 500 MHz) δ 7.33–7.17 (m, 5H), 4.88 (ddd, *J* = 10.4, 5.5, 5.0 Hz) and 4.65 (ddd, *J* = 10.0, 5.5, 4.7 Hz, total 1H), 3.73 (s) and 3.69 (s, total 3H), 3.42–2.87 (m, 2H), 2.69 (s) and 2.66 (s, total 3H), 1.35 (s) and 1.31 (s, total 9H); ^{13}C NMR (acetone-d_6, 125 MHz) δ 172.2 and 172.1, 156.2 and 155.2, 139.1 (d, *J* = 44.0 Hz) and 138.8 (d, *J* = 43.2 Hz), 130.0 and 129.9 (total 2C), 129.2 (d, *J* = 3.0 Hz) and 129.1 (d, *J* = 2.9 Hz, total 2C), 127.2 and 127.2, 80.1 and 79.9, 82.3 (d, *J* = 36.5Hz) and 80.7 (d, *J* = 36.7 Hz), 52.3 and 52.2, 36.1 and 35.5 (labeled), 33.0 and 32.7, 28.4 and 28.3 (total 3C); HRESIMS *m/z* 317.1557 $[M+Na]^+$ calcd. for $C_{16}H_{23}NO_4Na$, 317.1553 (^{13}C-containing); $[\alpha]_D^{20}$: –69.9 (*c* 0.20, MeOH) (**Supplementary Figs. 36** and **37**).

To a stirred solution of **S2** (57.0 mg, 0.194 mmol) in THF (1 mL) was added 1 M aq. NaOH (1 mL), and the resulting mixture was stirred for 1.5 h at room temperature. After being quenched with 0.5 M aq. sodium dihydrogen citrate (4 mL) and brine (1 mL), the mixture was extracted with EtOAc (2 × 5 mL). Combined organic layers were washed with brine (2 × 4 mL), dried over

Na_2SO_4 and concentrated *in vacuo*. The residue was dissolved in DCM (1 mL), and TFA (1 mL) was added to the solution. After being stirred for 1 h at room temperature, the solution was concentrated *in vacuo* to yield labeled *N*-methyl L-phenylalanine. This was reacted with isatoic anhydride (33.1 mg, 0.203 mmol) and DIEA (50 µL, 0.294 mmol) in dry DMSO (2 mL) for 3 h at 130 °C. After being cooled to room temperature, the solution was concentrated *in vacuo*. The residue was dissolved in EtOAc (10 mL) and washed with sat. aq. NH_4Cl (10 mL) and brine (10 mL), dried over Na_2SO_4 and concentrated *in vacuo*. The residue was chromatographed on SiO_2 (*n*-hexane:acetone = 2:1) followed by purification on RP–HPLC (Cosmosil 5C18-MSII, 250 × 10 mm) to yield ^{13}C-labeled **4** (6.4 mg, 0.023 mmol, 12%); ^{13}C-labeled **4** exists as two conformers in $CDCl_3$. ^{1}H NMR ($CDCl_3$, 500 MHz) δ 8.68 (s) and 8.38 (s, total 1H), 8.13 (d, *J* = 7.4 Hz) and 7.97 (d, *J* = 7.1 Hz, total 1H), 7.55 (d, *J* = 7.6, 7.6, 1.2 Hz) and 7.48 (d, *J* = 7.6, 7.6, 1.1 Hz, total 1H), 7.40–7.19 (m, 5H), 7.08–6.92 (m, 2H), 4.40–4.23 (m, 1H), 3.70–2.50 (m, 5H); ^{13}C NMR ($CDCl_3$, 125 MHz) δ 171.4, 169.8, 168.5, 166.0 (171.4 to 166.0 total 2C), 136.5 (d, *J* = 43.7 Hz) and 135.7 (d, *J* = 44.0 Hz), 135.5, 134.6, 132.9, 132.6, 132.0, 131.6, 129.2–128.8, 127.6, 127.5, 127.1, 127.0, 125.6, 125.4, 120.7, 120.1 (135.5 to 120.1 total 11C), 68.6 (d, *J* = 31.7 Hz) and 56.4 (d, *J* = 39.4 Hz), 41.3 and 39.8, 34.8 and 32.3 (labeled); HRESIMS *m/z* 282.1318 $[M+H]^+$ calcd. for $C_{17}H_{17}N_2O_2$, 282.1318 (^{13}C-containing); $[\alpha]_D^{20}$: –60.9 (*c* 0.51, MeOH) (**Supplementary Figs. 38** and **39**).

Preparation of ^{13}C-labeled cyclopenin 5 using AsqJ. Crude AsqJ was prepared from 1 L culture of *E. coli* harboring pKW5296 as described previously[25]. To a solution of crude AsqJ in 8 mL reaction buffer (0.1 M Tris-HCl (pH 7.4), 0.1 M NaCl, 0.1 mM $FeSO_4$, 2 mM ascorbic acid, 1 mM α-ketoglutaric acid) was added ^{13}C-labeled **4** (1.9 mg) and the reaction was continued overnight at room temperature. The mixture was extracted with EtOAc (3 × 8 mL) and combined organic layers were washed with brine (4 mL), dried over Na_2SO_4 and concentrated *in vacuo*. Crude product was purified by RP–HPLC (Cosmosil 5C18-MSII, 250 × 10 mm) to yield ^{13}C-labeled cyclopenin **5** (1.1 mg). ^{1}H NMR (DMSO-d_6, 500 MHz) δ 10.86 (br s, 1H), 7.54 (dd, *J* = 7.5, 7.5 Hz, 1H), 7.30 (dd, *J* = 7.0, 7.0 Hz, 1H), 7.22 (dd, *J* = 7.5, 7.5 Hz, 2H), 7.16 (d, *J* = 8.0 Hz, 1H), 7.08 (dd, *J* = 7.5, 7.5 Hz, 1H), 6.91 (d, *J* = 7.8 Hz, 1H), 6.63 (m, 2H), 4.37 (d, *J* = 182 Hz (coupled with ^{13}C) 1H), 3.07 (s, 3H); ^{13}C NMR (DMSO-d_6, 125 MHz) δ 166.0, 165.3, 135.2, 132.4, 131.0 (d, *J* = 56.9 Hz), 130.5, 128.7, 127.9 (d, *J* = 4.4 Hz, 2C), 126.4, 126.1 (d, *J* = 3.4 Hz,

2C), 124.3, 121.2, 70.6 (d, J = 27.6 Hz), 63.7 (labeled major conformer) and 61.2 (labeled minor conformer), 30.9; HRESIMS m/z 296.1109 $[M+H]^+$ calcd. for $C_{17}H_{15}N_2O_3$, 296.1111 (^{13}C-containing); $[\alpha]_D^{20}$: –214.3 (c 0.20, MeOH) (**Supplementary Figs. 25–27**).

Isolation of 6 and carbamothioate 9. To a mixture of 12 µM of AsqI in Tris buffer (100 mM Tris-HCl, 100 mM NaCl, pH 7.4), 2.9 mg (9.9 µmol) of **5** and 20 µmol of thiophenol **8** in DMF (300 µL) were added. After being incubated for 4.5 h at 30 °C, the reaction mixture was extracted with EtOAc (2 × 20 mL). Combined organic layers were washed with satd. aq. $NaHCO_3$ (2 × 10 mL) and brine (10 mL), dried over Na_2SO_4 and concentrated *in vacuo*. The residue was chromatographed on SiO_2 (*n*-hexane:acetone = 5:1, 1:1 and $CHCl_3$:MeOH = 1:1) followed by purification on RP–HPLC (Cosmosil 5C18-MSII, 250 × 10 mm) to yield **6**[26] (1.4 mg, 5.9 µmol) and carbamothioate **9** (1.0 mg, 6.0 µmol).

Isolation and chemical characterization of ^{13}C-labeled 6. To a mixture of 12 µM of AsqI in Tris buffer (100 mM Tris-HCl, 100 mM NaCl, pH 7.4), 0.9 mg (3 µmol) of ^{13}C-labeled **5** described earlier were added. After being incubated for 4.5 h at 30 °C, the reaction mixture was extracted with EtOAc (2 × 20 mL). Combined organic layers were washed with satd. aq. $NaHCO_3$ (2 × 10 mL) and brine (10 mL), dried over Na_2SO_4 and concentrated *in vacuo*. The residue was purified by using RP–HPLC (Cosmosil 5C18-MSII, 250 × 10 mm) to yield ^{13}C-labeled **6** (0.4 mg, 2 µmol). The NMR spectra of isolated ^{13}C-labeled **6** were shown earlier (**Supplementary Figs. 25–27**), and an observation of Jcc was described in **Supplementary Table 4** given earlier.

4. Supplementary References

1. Zou, Y. *et al.* Tandem prenyltransferases catalyze isoprenoid elongation and complexity generation in biosynthesis of quinolone alkaloids. *J. Am. Chem. Soc.* **137**, 4980–4983 (2015).
2. Finn, R.D. *et al.* The Pfam protein families database: towards a more sustainable future. *Nucleic Acids Res.* **44**, D279–D285 (2016).
3. Sievers, F. *et al.* Fast, scalable generation of high-quality protein multiple sequence alignments using Clustal Omega. *Mol. Syst. Biol.* **7**, 539 (2011).
4. Li, Y., Wang, Y., Jiang, H. & Deng, J. Crystal structure of *Manduca sexta* prophenoloxidase provides insights into the mechanism of type 3 copper enzymes. *Proc. Natl. Acad. Sci. USA* **106**, 17002–17006 (2009).
5. Magnus, K.A. *et al.* Crystallographic analysis of oxygenated and deoxygenated states of arthropod hemocyanin shows unusual differences. *Proteins* **19**, 302–309 (1994).
6. Hazes, B. *et al.* Crystal structure of deoxygenated *Limulus polyphemus* subunit II hemocyanin at 2.18 Å resolution: clues for a mechanism for allosteric regulation. *Protein Sci.* **2**, 597–619 (1993).
7. Cong, Y. *et al.* Structural mechanism of SDS-induced enzyme activity of scorpion hemocyanin revealed by electron cryomicroscopy. *Structure* **17**, 749–758 (2009).
8. Masuda, T., Momoji, K., Hirata, T. & Mikami, B. The crystal structure of a crustacean prophenoloxidase provides a clue to understanding the functionality of the type 3 copper proteins. *FEBS J.* **281**, 2659–2673 (2014).
9. Hu, Y., Wang, Y., Deng, J. & Jiang, H. The structure of a prophenoloxidase (PPO) from *Anopheles gambiae* provides new insights into the mechanism of PPO activation. *BMC Biol.* **14**, 2 (2016).
10. Volbeda, A. & Hol, W.G. Crystal structure of hexameric haemocyanin from *Panulirus interruptus* refined at 3.2 Å resolution. *J. Mol. Biol.* **209**, 249–279 (1989).
11. Bracken, A., Pocker, A. & Raistrick, H. Studies in the biochemistry of microorganisms. 93. Cyclopenin, a nitrogen-containing metabolic product of *Penicillium cyclopium* Westling. *Biochem. J.* **57**, 587–595 (1954).
12. Zolan, M.E. & Pukkila, P.J. Inheritance of DNA methylation in *Coprinus cinereus*. *Mol. Cell Biol.* **6**, 195–200 (1986).
13. Ohnishi, K. & Ono, B. Inverted repeat of a large segment unveiled on the right arm of *Saccharomyces cerevisiae* chromosome II. *Yeast* **22**, 321–336 (2005).
14. Galagan, J.E. *et al.* Sequencing of *Aspergillus nidulans* and comparative analysis with *A. fumigatus* and *A. oryzae*. *Nature* **438**, 1105–1115 (2005).
15. Johnson, M. *et al.* NCBI BLAST: a better web interface. *Nucleic Acids Res.* **36**, W5–W9 (2008).
16. Marchler-Bauer, A. *et al.* CDD: a Conserved Domain Database for the functional annotation of proteins. *Nucleic Acids Res.* **39**, D225–D229 (2011).

17. Tsunematsu, Y. *et al*. Distinct mechanisms for spiro-carbon formation reveal biosynthetic pathway crosstalk. *Nat. Chem. Biol.* **9**, 818–825 (2013).

18. Blumhoff, M., Steiger, M.G., Marx, H., Mattanovich, D. & Sauer, M. Six novel constitutive promoters for metabolic engineering of *Aspergillus niger*. *Appl. Microbiol. Biotechnol.* **97**, 259–267 (2013).

19. Cerqueira, G.C. *et al.* The *Aspergillus* Genome Database: multispecies curation and incorporation of RNA-Seq data to improve structural gene annotations. *Nucleic Acids Res.* **42**, D705–D710 (2014).

20. Kabsch, W. XDS. *Acta Crystallogr. D.* **66**, 125-132 (2010).

21. Evans, P.R. & Murshudov, G.N. How good are my data and what is the resolution? *Acta Crystallogr. D.* **69**, 1204–1214 (2013).

22. Eswar, N. *et al.* Comparative protein structure modeling using MODELLER. *Curr. Protoc. Protein Sci.* **Chapter 2**, Unit 2.9 (2007).

23. Xu, D. & Zhang, Y. Improving the physical realism and structural accuracy of protein models by a two-step atomic-level energy minimization. *Biophys J* **101**, 2525–2534 (2011).

24. Gottlieb, H.E., Kotlyar, V. & Nudelman, A. NMR chemical shifts of common laboratory solvents as trace impurities. *J. Org. Chem.* **62**, 7512–7515 (1997).

25. Ishikawa, N. *et al.* Non-heme dioxygenase catalyzes atypical oxidations of 6,7-bicyclic systems to form the 6,6-quinolone core of viridicatin-type fungal alkaloids. *Angew. Chem. Int. Ed. Engl.* **53**, 12880–12884 (2014).

26. Cunningham, K.G. & Freeman, G.G. The isolation and some chemical properties of viridicatin, a metabolic product of *Penicillium viridicatum* Westling. *Biochem. J.* **53**, 328–332 (1953).
