# Supplementary material for: Enzymatic one-step ring contraction for quinolone biosynthesis
Source: Nat Commun. 2018 Jul 19;9:2826. doi: 10.1038/s41467-018-05221-5 (PMC6053404; doi:10.1038/s41467-018-05221-5)
Supplement: Supplementary file 3 — Description of Additional Supplementary Files [file 41467_2018_5221_MOESM3_ESM.pdf]

Description of Additional Supplementary Files

File Name: Supplementary Data 1
Description: Cartesian coordinates, energies and vibrational frequencies of (–)-cyclopenin **5**, computed transition states (TSs), reaction intermediates (Ints) and products related to the uncatalyzed pathway. All the following geometries, frequencies and energy correction were obtained using M06-2X/6-31G(d). Single-point SCF energies were calculated at the M06-2X/6-311+G(d,p)/SMD(Et_2O) level of theory.

File Name: Supplementary Data 2
Description: Cartesian coordinates, energies and vibrational frequencies of (–)-cyclopenin **5**, computed transition states (TSs), reaction intermediates (Ints) and products related to the methylammonium (MA)-catalyzed pathways. All the following geometries, frequencies and energy correction were obtained using M06-2X/6-31G(d). Single-point SCF energies were calculated at the M06-2X/6-311+G(d,p)/SMD(Et_2O) level of theory.

File Name: Supplementary Data 3
Description: Cartesian coordinates, energies and vibrational frequencies of (–)-4'-methoxycyclopenin **2**, computed transition states (TSs), reaction intermediates (Ints) and products related to the methylammonium (MA)-catalyzed pathways. All the following geometries, frequencies and energy correction were obtained using M06-2X/6-31G(d). Single-point SCF energies were calculated at the M06-2X/6-311+G(d,p)/SMD(Et_2O) level of theory.

File Name: Supplementary Data 4
Description: Cartesian coordinates, energies and vibrational frequencies of (–)-cyclopenin **5**, computed transition states (TSs), reaction intermediates (Ints) and products related to the pathways catalyzed by the Zn^{2+} ion with two imidazole molecules (denoted by "Zn + 2 His"). All the following geometries, frequencies and energy correction were obtained using B3LYP/6-31G(d)/LANL2DZ(Zn)/SMD(Et_2O). Single-point SCF energies were calculated at the M06-2X/6-311+G(d,p)/SMD(Et_2O) level of theory.
