# Supplementary material for: Enzymatic one-step ring contraction for quinolone biosynthesis
Source: Nat Commun. 2018 Jul 19;9:2826. doi: 10.1038/s41467-018-05221-5 (PMC6053404; doi:10.1038/s41467-018-05221-5)
Supplement: Supplementary file 4 — Supplementary Data 1 [file 41467_2018_5221_MOESM4_ESM.docx]

**Supplementary Data Set 1.**

**(–)-Cyclopenin 5 (uncatalyzed)**

C 2.34084 -3.01918 -0.48519

C 2.75866 -1.71514 -0.70197

C 2.11486 -0.63977 -0.07600

C 1.04003 -0.89660 0.79190

C 0.63754 -2.21937 1.00169

C 1.26645 -3.27792 0.36596

H 2.85424 -3.83434 -0.98547

H 3.59119 -1.51021 -1.36957

H -0.18064 -2.38367 1.69543

H 0.93427 -4.29595 0.53927

C 0.31847 0.13200 1.61584

C 0.42649 1.60582 -0.31971

C 1.89474 1.78016 -0.63393

O -0.09626 -0.12793 2.73164

O 2.38381 2.78229 -1.09878

N 2.62616 0.65436 -0.32427

H 3.59682 0.71474 -0.61051

N 0.15203 1.37276 1.06201

C -0.49282 2.40634 1.86142

H -0.41627 3.35523 1.33091

H -1.54597 2.15948 2.02831

H 0.00468 2.47297 2.83038

C -0.47353 1.07693 -1.37786

H -0.01961 0.88889 -2.35196

C -1.65703 0.25416 -1.01525

C -1.64096 -1.11255 -1.29987

C -2.75098 0.81566 -0.35896

C -2.71290 -1.91432 -0.92311

H -0.77739 -1.54959 -1.79751

C -3.82507 0.01136 0.01187

H -2.75363 1.88194 -0.15348

C -3.80666 -1.35288 -0.26683

H -2.69327 -2.97794 -1.13978

H -4.67754 0.45168 0.51960

H -4.64403 -1.97836 0.02625

O -0.45617 2.44652 -0.99112

SCF energy: -991.315784 hartree

zero-point correction: +0.284850 hartree

enthalpy correction: +0.302605 hartree

free energy correction: +0.239943 hartree

quasiharmonic free energy correction: +0.242240 hartree

**TS 1 (uncatalyzed)**

C -1.57303 3.45393 -0.06379

C -2.36170 2.38473 -0.42314

C -1.94788 1.06933 -0.12779

C -0.72876 0.84827 0.59062

C 0.03280 1.99852 0.96317

C -0.34762 3.26560 0.61414

H -1.89704 4.45880 -0.31792

H -3.29106 2.53669 -0.96408

H 0.93015 1.82122 1.54858

H 0.25900 4.12170 0.88686

C -0.57477 -0.35080 1.54948

C -0.66657 -1.46107 -0.59334

C -2.16007 -1.25691 -0.95798

O -0.36443 -0.16411 2.73511

O -2.86622 -2.05865 -1.50614

N -2.67790 0.01374 -0.60816

H -3.59998 0.19866 -0.99125

N -0.67690 -1.52687 0.91763

C -0.55121 -2.79925 1.58903

H -0.26012 -3.52574 0.82465

H 0.21652 -2.72878 2.36488

H -1.49426 -3.10387 2.05278

C 0.13595 -0.20942 -0.93025

H -0.20125 0.32460 -1.82188

C 1.58642 -0.13140 -0.71097

C 2.26409 0.98757 -1.21426

C 2.29642 -1.09183 0.02495

C 3.61852 1.16562 -0.96595

H 1.71387 1.72862 -1.78964

C 3.65326 -0.91400 0.26187

H 1.78768 -1.99138 0.34907

C 4.31398 0.21439 -0.22216

H 4.13217 2.03793 -1.35724

H 4.20134 -1.66565 0.82063

H 5.37451 0.34607 -0.03042

O -0.06022 -2.40308 -1.23437

One imaginary frequency: -430.64 cm^-1^.

SCF energy: -991.240483 hartree

zero-point correction: +0.281375 hartree

enthalpy correction: +0.299828 hartree

free energy correction: +0.236302 hartree

quasiharmonic free energy correction: +0.237906 hartree

**Viridicatin 6 tautomer (uncatalyzed)**

C -2.71957 -2.71730 -0.23158

C -3.14289 -1.39974 -0.10192

C -2.20757 -0.38937 0.11278

C -0.84110 -0.68727 0.20835

C -0.43621 -2.01093 0.06474

C -1.36515 -3.02552 -0.15526

H -3.45247 -3.49984 -0.39949

H -4.19736 -1.14751 -0.17506

H 0.62192 -2.24480 0.13176

H -1.02889 -4.05119 -0.26304

C -0.34556 1.73769 -0.10940

C -1.84774 2.05242 0.05808

O -2.27942 3.18029 0.02651

N -2.63514 0.94373 0.24713

H -3.63012 1.13214 0.29913

C 0.12632 0.44136 0.54431

H 0.00633 0.63770 1.62535

C 1.57788 0.14081 0.26885

C 2.47796 -0.02523 1.31905

C 2.03219 0.00312 -1.04580

C 3.81572 -0.32316 1.06636

H 2.13064 0.07999 2.34407

C 3.36551 -0.29331 -1.30039

H 1.33553 0.13448 -1.86940

C 4.26092 -0.45734 -0.24411

H 4.50716 -0.44821 1.89384

H 3.70947 -0.39248 -2.32525

H 5.30282 -0.68665 -0.44523

O 0.35900 2.52006 -0.68537

SCF energy: -783.350377 hartree

zero-point correction: +0.227951 hartree

enthalpy correction: +0.242566 hartree

free energy correction: +0.186139 hartree

quasiharmonic free energy correction: +0.188504 hartree

**Viridicatin 6 (uncatalyzed)**

C 3.01862 -2.54714 -0.18175

C 3.30999 -1.20192 -0.04648

C 2.27108 -0.26659 0.00394

C 0.92090 -0.66773 -0.06448

C 0.66114 -2.04094 -0.22465

C 1.68784 -2.96682 -0.27972

H 3.82627 -3.27078 -0.22244

H 4.33912 -0.85786 0.01464

C -0.12968 0.34306 -0.00399

H -0.36802 -2.37040 -0.31681

H 1.45797 -4.01955 -0.40580

C 0.23151 1.65058 0.05618

C 1.63530 2.08601 0.12492

O 1.91963 3.27804 0.20794

N 2.56223 1.08639 0.11032

H 3.52865 1.38728 0.16468

O -0.64273 2.67054 0.09854

H -0.09102 3.47557 0.18096

C -1.56716 -0.03270 0.00354

C -2.44992 0.51978 -0.92898

C -2.06335 -0.92340 0.96214

C -3.79793 0.17724 -0.91182

H -2.07169 1.22361 -1.66365

C -3.41220 -1.26277 0.98084

H -1.38419 -1.34195 1.70031

C -4.28206 -0.71584 0.04074

H -4.47287 0.61003 -1.64382

H -3.78473 -1.95222 1.73229

H -5.33464 -0.98184 0.05316

SCF energy: -783.373147 hartree

zero-point correction: +0.229539 hartree

enthalpy correction: +0.243782 hartree

free energy correction: +0.189354 hartree

quasiharmonic free energy correction: +0.190364 hartree

**Methyl isocyanate 7 (uncatalyzed)**

O 1.90234 0.13724 0.00002

C 0.74145 -0.05004 0.00002

N -0.41243 -0.37107 -0.00008

C -1.74656 0.15661 0.00001

H -2.45517 -0.67236 -0.00738

H -1.92512 0.76007 0.89438

H -1.92073 0.77244 -0.88677

SCF energy: -207.967497 hartree

zero-point correction: +0.051452 hartree

enthalpy correction: +0.056303 hartree

free energy correction: +0.025516 hartree

quasiharmonic free energy correction: +0.025516 hartree
