# Supplementary material for: Enzymatic one-step ring contraction for quinolone biosynthesis
Source: Nat Commun. 2018 Jul 19;9:2826. doi: 10.1038/s41467-018-05221-5 (PMC6053404; doi:10.1038/s41467-018-05221-5)
Supplement: Supplementary file 5 — Supplementary Data 2 [file 41467_2018_5221_MOESM5_ESM.docx]

**Supplementary Data Set 2.**

**(–)-Cyclopenin 5 (MA)**

C -2.76933 -2.95699 -1.18533

C -1.39804 -3.07569 -1.00796

C -0.72568 -2.24066 -0.11308

C -1.43812 -1.28194 0.62438

C -2.82075 -1.18474 0.43512

C -3.48592 -2.00201 -0.46551

H -3.27619 -3.61283 -1.88545

H -0.83585 -3.81899 -1.56637

H -3.35581 -0.45343 1.03210

H -4.55829 -1.90733 -0.59703

C -0.87757 -0.38287 1.68982

C 1.07943 -0.11688 0.28544

C 1.62567 -1.50696 0.08490

O -1.53072 -0.05571 2.65754

O 2.83495 -1.75072 0.00614

N 0.68081 -2.45421 0.01732

N 0.42095 0.05705 1.53130

C 0.97889 0.90553 2.57978

H 2.01837 1.12837 2.33766

H 0.41068 1.83670 2.65608

H 0.92211 0.38423 3.53662

C 0.72143 0.69149 -0.90957

H 0.90679 0.22904 -1.88048

C -0.36962 1.69399 -0.83693

C -1.54818 1.43087 -1.53799

C -0.25855 2.84070 -0.05233

C -2.61711 2.31519 -1.44836

H -1.63119 0.52996 -2.14264

C -1.32874 3.72697 0.02789

H 0.66766 3.03953 0.47880

C -2.50715 3.46364 -0.66644

H -3.53430 2.10984 -1.99056

H -1.24328 4.62424 0.63209

H -3.34041 4.15557 -0.60038

O 1.92104 0.91754 -0.14865

N 4.60657 0.22303 -0.13517

H 4.21957 1.08833 0.25135

H 5.49552 0.01659 0.32758

C 4.75707 0.32849 -1.61601

H 3.77681 0.57117 -2.02352

H 5.47595 1.10897 -1.86057

H 5.09392 -0.63509 -1.99524

H 3.89861 -0.55253 0.06751

H 1.02244 -3.40108 -0.11888

SCF energy: -1087.613958 hartree

zero-point correction: +0.366341 hartree

enthalpy correction: +0.389451 hartree

free energy correction: +0.313457 hartree

quasiharmonic free energy correction: +0.318279 hartree

**TS_MA_1 (MA)**

C -3.30095 -2.42658 -0.80924

C -1.97529 -2.69876 -1.09936

C -0.94996 -2.13726 -0.32381

C -1.27571 -1.30034 0.76297

C -2.62597 -1.05107 1.04614

C -3.63148 -1.59000 0.26441

H -4.08336 -2.86939 -1.41696

H -1.71217 -3.35079 -1.92724

H -2.85400 -0.43929 1.91308

H -4.67128 -1.38582 0.49563

C -0.28048 -0.73457 1.74455

C 1.07615 -0.16413 -0.24167

C 1.43931 -1.60113 -0.67310

O -0.53451 -0.70682 2.93220

O 2.57212 -1.93042 -0.95438

N 0.38273 -2.46658 -0.65312

N 0.87403 -0.22115 1.20876

C 1.99293 0.14400 2.06352

H 2.85574 -0.49972 1.85555

H 2.27996 1.18612 1.90057

H 1.67324 0.00093 3.09492

C -0.13137 0.35556 -0.99088

H -0.33879 -0.13184 -1.94418

C -0.84089 1.52845 -0.70513

C -1.93752 1.86097 -1.54605

C -0.52096 2.36595 0.39977

C -2.70934 2.97293 -1.27210

H -2.17092 1.22067 -2.39258

C -1.28564 3.48553 0.64737

H 0.34029 2.13191 1.01303

C -2.38027 3.78042 -0.17801

H -3.55518 3.22560 -1.90132

H -1.04326 4.13879 1.47798

H -2.98071 4.66054 0.03270

O 1.98974 0.76546 -0.62591

H 3.06703 0.41003 -0.77910

N 4.40778 0.18926 -0.89610

H 4.55214 -0.75780 -0.54680

H 4.65208 0.16530 -1.88600

C 5.22071 1.18324 -0.17257

H 4.92041 1.18584 0.87668

H 6.29058 0.97145 -0.23679

H 5.02421 2.17122 -0.59107

H 0.58380 -3.40952 -0.97093

One imaginary frequency: -237.40 cm^-1^.

SCF energy: -1087.561319 hartree

zero-point correction: +0.359526 hartree

enthalpy correction: +0.382672 hartree

free energy correction: +0.307221 hartree

quasiharmonic free energy correction: +0.311306 hartree

**Int_MA_1 (MA)**

C -3.33516 -2.44543 -0.73722

C -2.01857 -2.73059 -1.04823

C -0.97384 -2.14833 -0.31190

C -1.27502 -1.28092 0.76003

C -2.62128 -1.02088 1.06532

C -3.64250 -1.57501 0.31940

H -4.13198 -2.90330 -1.31437

H -1.77582 -3.40549 -1.86390

H -2.82937 -0.38921 1.92308

H -4.67711 -1.36221 0.56545

C -0.26031 -0.73046 1.73192

C 1.05142 -0.16548 -0.27817

C 1.40355 -1.60131 -0.72526

O -0.49690 -0.71218 2.92286

O 2.52359 -1.92971 -1.04659

N 0.34777 -2.47283 -0.67146

N 0.88816 -0.22782 1.17842

C 2.03307 0.11064 2.00938

H 2.87941 -0.54624 1.77625

H 2.33447 1.14883 1.84799

H 1.73467 -0.03503 3.04688

C -0.21091 0.32968 -0.96790

H -0.49234 -0.19101 -1.88327

C -0.87234 1.53026 -0.68961

C -2.00657 1.86235 -1.48111

C -0.46118 2.40530 0.35519

C -2.72029 3.01470 -1.22107

H -2.31049 1.19067 -2.27958

C -1.17169 3.56264 0.59054

H 0.42414 2.16619 0.93134

C -2.30052 3.85955 -0.18707

H -3.59106 3.27009 -1.81403

H -0.86074 4.24418 1.37430

H -2.85719 4.77031 0.01362

O 1.96694 0.75970 -0.67319

H 2.99217 0.40190 -0.76277

N 4.43185 0.14561 -0.84628

H 4.56261 -0.80280 -0.49744

H 4.68158 0.11295 -1.83417

C 5.27193 1.11632 -0.12642

H 4.97885 1.12689 0.92536

H 6.33863 0.88487 -0.19231

H 5.09811 2.11066 -0.54078

H 0.53923 -3.40765 -1.01811

SCF energy: -1087.560971 hartree

zero-point correction: +0.361527 hartree

enthalpy correction: +0.385196 hartree

free energy correction: +0.308294 hartree

quasiharmonic free energy correction: +0.312932 hartree

**TS_MA_2 (MA)**

C -3.32499 -2.50258 -0.68655

C -2.01160 -2.80626 -0.98523

C -0.96401 -2.17548 -0.29143

C -1.26257 -1.24347 0.73024

C -2.61399 -0.97030 1.02505

C -3.63338 -1.56519 0.31573

H -4.12360 -2.99503 -1.23193

H -1.77264 -3.52661 -1.76213

H -2.82014 -0.29238 1.84738

H -4.66826 -1.34050 0.54924

C -0.25144 -0.71053 1.72117

C 1.03823 -0.16591 -0.28589

C 1.39758 -1.59843 -0.73765

O -0.50209 -0.69725 2.90856

O 2.50980 -1.91936 -1.08722

N 0.35252 -2.48852 -0.65487

N 0.90518 -0.23206 1.17333

C 2.06206 0.08590 1.99333

H 2.89184 -0.59154 1.75890

H 2.38382 1.11617 1.82069

H 1.76795 -0.04481 3.03433

C -0.27176 0.29390 -0.91951

H -0.58291 -0.23482 -1.82011

C -0.90320 1.52151 -0.65996

C -2.04937 1.85192 -1.43115

C -0.44397 2.42249 0.33907

C -2.72504 3.03374 -1.20077

H -2.39287 1.15819 -2.19398

C -1.11846 3.60751 0.54754

H 0.44807 2.18199 0.90429

C -2.25769 3.90697 -0.21224

H -3.60323 3.28892 -1.78284

H -0.76890 4.30865 1.29701

H -2.78411 4.84004 -0.03424

O 1.93497 0.76683 -0.69923

H 2.95386 0.42617 -0.78470

N 4.42238 0.19275 -0.90208

H 4.56197 -0.79432 -0.69223

H 4.66576 0.30690 -1.88541

C 5.26592 1.05535 -0.05921

H 5.01964 0.87744 0.98957

H 6.33512 0.87544 -0.20262

H 5.04789 2.09945 -0.29033

H 0.54492 -3.41324 -1.02775

One imaginary frequency: -83.51 cm^-1^.

SCF energy: -1087.560331 hartree

zero-point correction: +0.361557 hartree

enthalpy correction: +0.384519 hartree

free energy correction: +0.309147 hartree

quasiharmonic free energy correction: +0.313728 hartree

**Int_MA_2 (MA)**

C -3.10556 -2.97587 -0.05143

C -1.83613 -3.24243 -0.50788

C -0.89844 -2.19713 -0.53563

C -1.23394 -0.86456 0.00988

C -2.64846 -0.64849 0.33951

C -3.53098 -1.67248 0.35660

H -3.82602 -3.78901 -0.02350

H -1.56083 -4.23329 -0.85419

H -2.92978 0.35580 0.64301

H -4.55972 -1.52217 0.66209

C -0.37983 -0.86890 1.42265

C 0.98309 -0.07130 -0.36120

C 1.38025 -1.40066 -1.03817

O -0.81556 -1.25229 2.46889

O 2.45111 -1.67888 -1.49856

N 0.34463 -2.38060 -1.01890

N 0.83537 -0.41780 1.07601

C 1.96483 -0.36779 1.98783

H 2.70414 -1.13494 1.72992

H 2.42669 0.62063 1.92613

H 1.59340 -0.54729 2.99733

C -0.46843 0.24825 -0.77743

H -0.58482 0.05756 -1.85048

C -0.94903 1.64932 -0.48983

C -1.87076 2.20571 -1.38171

C -0.56907 2.38137 0.63950

C -2.41185 3.46616 -1.15260

H -2.16490 1.64737 -2.26775

C -1.10931 3.64395 0.86613

H 0.16462 1.98483 1.33268

C -2.03307 4.18713 -0.02349

H -3.12210 3.88503 -1.85780

H -0.80260 4.20745 1.74125

H -2.45045 5.17206 0.15866

O 1.84556 0.92881 -0.59917

H 2.84268 0.63680 -0.59658

N 4.41906 0.39706 -0.65916

H 4.57774 -0.58339 -0.88468

H 4.71014 0.92147 -1.48370

C 5.22681 0.80876 0.50038

H 4.94800 0.20305 1.36543

H 6.30320 0.70037 0.33554

H 5.01007 1.85368 0.73071

H 0.61762 -3.29390 -1.37768

SCF energy: -1087.591788 hartree

zero-point correction: +0.364202 hartree

enthalpy correction: +0.387191 hartree

free energy correction: +0.312376 hartree

quasiharmonic free energy correction: +0.316367 hartree

**TS_MA_3 (MA)**

C -3.07223 -2.83981 -0.49619

C -1.74191 -3.11293 -0.77202

C -0.82998 -2.05682 -0.81792

C -1.26067 -0.72019 -0.59044

C -2.63349 -0.47297 -0.39045

C -3.52425 -1.52348 -0.30886

H -3.77680 -3.66270 -0.42972

H -1.40247 -4.13288 -0.92450

H -2.96258 0.55315 -0.25969

H -4.57309 -1.33626 -0.10854

C -0.70373 -1.16299 1.53516

C 1.01343 -0.12912 0.00900

C 1.52889 -1.43264 -0.64851

O -1.54535 -1.67406 2.15136

O 2.70448 -1.69090 -0.76185

N 0.52749 -2.28983 -1.04364

N 0.46210 -0.64793 1.39511

C 1.39932 -0.62075 2.53556

H 2.23003 -1.29821 2.32165

H 1.76472 0.40088 2.64006

H 0.87806 -0.93905 3.43820

C -0.21754 0.36988 -0.75695

H 0.09137 0.37296 -1.81343

C -0.70103 1.76149 -0.41132

C -1.33861 2.49011 -1.41883

C -0.60074 2.31745 0.86574

C -1.87501 3.74673 -1.15780

H -1.41830 2.06687 -2.41785

C -1.13236 3.57741 1.12629

H -0.08471 1.78757 1.65936

C -1.77406 4.29262 0.11899

H -2.36549 4.29923 -1.95251

H -1.03887 4.00336 2.12004

H -2.18727 5.27438 0.32600

O 1.94616 0.78458 0.21690

H 2.97677 0.46167 0.25285

N 4.44996 0.33061 0.18256

H 4.96648 0.89018 0.85929

H 4.71813 -0.64241 0.31595

C 4.78121 0.75277 -1.19229

H 4.43456 1.77745 -1.33773

H 5.85211 0.70078 -1.40503

H 4.24579 0.10013 -1.88354

H 0.82892 -3.20460 -1.36535

One imaginary frequency: -94.45 cm^-1^.

SCF energy: -1087.583985 hartree

zero-point correction: +0.361820 hartree

enthalpy correction: +0.384579 hartree

free energy correction: +0.310524 hartree

quasiharmonic free energy correction: +0.314174 hartree

**Viridicatin 6 tautomer (MA)**

C -4.38876 -0.59433 0.33128

C -3.30244 -1.24409 0.90539

C -2.02414 -1.00125 0.41123

C -1.81169 -0.11913 -0.65085

C -2.91321 0.52037 -1.21330

C -4.19699 0.28632 -0.73076

H -5.38618 -0.78134 0.71418

H -3.44527 -1.94002 1.72733

H -2.75521 1.21543 -2.03339

H -5.04454 0.79012 -1.18204

C 0.54081 -0.94661 -0.81806

C 0.32261 -1.71278 0.49916

O 1.24635 -2.33782 1.02317

N -0.91637 -1.66254 1.00303

C -0.40931 0.18177 -1.11576

H -0.38471 0.36804 -2.19396

O 1.52283 -1.21776 -1.47244

C 0.24140 1.37752 -0.39087

C 1.40523 1.92750 -0.93886

C -0.26680 1.88719 0.80282

C 2.04191 2.99036 -0.30801

H 1.79901 1.52968 -1.87181

C 0.37678 2.95090 1.43445

H -1.17701 1.47765 1.23118

C 1.52784 3.50332 0.88307

H 2.93028 3.42849 -0.75196

H -0.03043 3.35054 2.35750

H 2.02069 4.33614 1.37385

N 3.66401 -2.29695 -0.15912

H 3.53291 -2.34028 -1.17364

H 2.70018 -2.44095 0.26126

C 4.16106 -0.95259 0.25953

H 5.14415 -0.77449 -0.17349

H 3.44696 -0.20894 -0.09509

H 4.21313 -0.93123 1.34704

H 4.29384 -3.04830 0.13309

H -1.07047 -2.19629 1.85418

SCF energy: -879.654648 hartree

zero-point correction: +0.310812 hartree

enthalpy correction: +0.329593 hartree

free energy correction: +0.263960 hartree

quasiharmonic free energy correction: +0.267152 hartree

**Viridicatin 6 (MA)**

C -0.74657 4.16220 -0.12146

C 0.45812 3.49182 -0.06297

C 0.46112 2.09211 -0.03864

C -0.73248 1.34564 -0.05885

C -1.94435 2.06405 -0.14132

C -1.95113 3.44337 -0.16889

H -0.75803 5.24660 -0.14210

H 1.39864 4.03594 -0.04160

H -2.87646 1.51332 -0.19633

H -2.89382 3.97533 -0.23580

C 0.56008 -0.70439 -0.04051

C 1.78662 0.06301 -0.01732

O 2.90057 -0.53304 0.01186

N 1.66965 1.40389 -0.00718

C -0.66664 -0.10232 -0.03133

O 0.71241 -2.04392 -0.03277

C -1.89392 -0.93569 0.00609

C -2.84207 -0.75854 1.01997

C -2.09900 -1.92388 -0.96128

C -3.98074 -1.55650 1.06028

H -2.67800 -0.00413 1.78499

C -3.24352 -2.71209 -0.92439

H -1.35900 -2.06960 -1.74183

C -4.18497 -2.53083 0.08593

H -4.70804 -1.41792 1.85362

H -3.39904 -3.47111 -1.68409

H -5.07604 -3.14957 0.11544

H 1.66860 -2.21677 0.01169

N 5.46124 -0.67444 0.10815

H 4.43108 -0.35883 0.06232

H 5.95579 -0.34868 -0.72657

C 5.46505 -2.16759 0.19678

H 4.90204 -2.44994 1.08480

H 4.96803 -2.55516 -0.69107

H 6.48869 -2.53392 0.25603

H 5.91400 -0.25108 0.92238

H 2.52782 1.94292 0.01612

SCF energy: -879.667720 hartree

zero-point correction: +0.310140 hartree

enthalpy correction: +0.329303 hartree

free energy correction: +0.261331 hartree

quasiharmonic free energy correction: +0.266023 hartree
