# Supplementary material for: Enzymatic one-step ring contraction for quinolone biosynthesis
Source: Nat Commun. 2018 Jul 19;9:2826. doi: 10.1038/s41467-018-05221-5 (PMC6053404; doi:10.1038/s41467-018-05221-5)
Supplement: Supplementary file 6 — Supplementary Data 3 [file 41467_2018_5221_MOESM6_ESM.docx]

**Supplementary Data Set 3.**

**4'-Methoxycylopenin 2 (MA)**

C 1.58015 3.64178 -1.07725

C 0.21546 3.39208 -1.04815

C -0.31397 2.45711 -0.15553

C 0.53148 1.77394 0.73205

C 1.90297 2.04919 0.69179

C 2.43162 2.96207 -0.20765

H 1.97628 4.36606 -1.78106

H -0.45271 3.91741 -1.72492

H 2.53830 1.52376 1.39724

H 3.49938 3.14967 -0.22698

C 0.11046 0.80052 1.79739

C -1.52063 -0.05820 0.21680

C -2.38865 1.11768 -0.15091

O 0.71589 0.70560 2.84327

O -3.60157 1.01570 -0.36798

N -1.73158 2.28340 -0.19281

N -1.00288 0.02447 1.53749

C -1.46420 -0.91085 2.55658

H -2.53882 -1.07011 2.43897

H -0.93831 -1.86733 2.48287

H -1.26434 -0.46833 3.53155

C -0.82956 -0.82070 -0.85440

H -1.00902 -0.49388 -1.87994

C 0.46307 -1.49445 -0.56408

C 1.62627 -0.86700 -0.99233

C 0.51719 -2.68813 0.15743

C 2.87236 -1.41909 -0.67965

H 1.60161 0.06600 -1.54987

C 1.75932 -3.24066 0.44420

H -0.39870 -3.17689 0.47227

C 2.93751 -2.61700 0.03858

H 1.81994 -4.17317 0.99602

H 3.89150 -3.06765 0.28425

O -2.01206 -1.30588 -0.19662

N -4.76336 -1.37281 -0.41311

H -4.18431 -2.05239 0.08819

H -5.70922 -1.38419 -0.02326

C -4.75634 -1.65461 -1.87826

H -3.71697 -1.64682 -2.20261

H -5.20947 -2.62578 -2.07081

H -5.31245 -0.86520 -2.38157

H -4.31336 -0.41381 -0.26070

H -2.29410 3.09349 -0.43559

O 3.94379 -0.71639 -1.10703

C 5.23015 -1.23739 -0.82209

H 5.39313 -1.31550 0.25883

H 5.36909 -2.21999 -1.28623

H 5.94092 -0.53070 -1.24784

SCF energy: -1202.128574 hartree

zero-point correction: +0.400152 hartree

enthalpy correction: +0.425568 hartree

free energy correction: +0.344988 hartree

quasiharmonic free energy correction: +0.349991 hartree

**TS_MA_1 (MA)**

C 0.12711 4.05519 -1.25951

C -1.03703 3.30398 -1.29490

C -1.25982 2.29270 -0.35311

C -0.29814 2.04013 0.64143

C 0.86692 2.81584 0.66276

C 1.09001 3.80766 -0.27908

H 0.28263 4.83629 -1.99636

H -1.79279 3.49293 -2.05198

H 1.57840 2.62841 1.46050

H 1.99878 4.39860 -0.24441

C -0.44884 1.05342 1.76807

C -1.42210 -0.53659 0.11578

C -2.68668 0.26319 -0.26576

O 0.01966 1.29769 2.86568

O -3.78034 -0.26362 -0.37149

N -2.49115 1.59869 -0.42851

N -1.08697 -0.12885 1.48605

C -1.45055 -1.04003 2.56117

H -2.52481 -1.24946 2.52651

H -0.90898 -1.98634 2.47777

H -1.20399 -0.55009 3.50156

C -0.31470 -0.37089 -0.89097

H -0.65878 -0.13654 -1.89929

C 1.02968 -0.67840 -0.74262

C 1.88271 -0.53937 -1.88305

C 1.61422 -1.10129 0.49064

C 3.22553 -0.76756 -1.79413

H 1.44811 -0.22937 -2.82936

C 2.95833 -1.35060 0.58454

H 0.98230 -1.24540 1.35874

C 3.78188 -1.17644 -0.55618

H 3.89235 -0.65648 -2.64142

H 3.38072 -1.68118 1.52514

O -1.56787 -1.87339 -0.03717

H -2.73963 -2.39111 0.00042

N -3.80138 -2.97074 -0.08385

H -4.49299 -2.32965 0.30611

H -3.31387 2.12285 -0.70863

O 5.08208 -1.38037 -0.56187

C 5.74701 -1.80108 0.63279

H 5.62659 -1.05105 1.41884

H 5.36111 -2.76946 0.96198

H 6.79641 -1.89120 0.36176

C -4.06069 -3.18851 -1.52456

H -4.04618 -2.21127 -2.00634

H -3.26262 -3.80984 -1.93154

H -5.02587 -3.66959 -1.68794

H -3.81775 -3.84516 0.44099

One imaginary frequency: -177.32 cm^-1^.

SCF energy: -1202.091062 hartree

zero-point correction: +0.394011 hartree

enthalpy correction: +0.419435 hartree

free energy correction: +0.338754 hartree

quasiharmonic free energy correction: +0.343839 hartree

**Int_MA_1 (MA)**

C 0.00442 4.39838 -0.55049

C -1.08390 3.61977 -0.90838

C -1.35149 2.41986 -0.23523

C -0.51160 2.01086 0.81770

C 0.57617 2.82095 1.17025

C 0.84813 3.99444 0.48922

H 0.19569 5.32598 -1.08009

H -1.74370 3.93063 -1.71334

H 1.18055 2.50976 2.01616

H 1.69510 4.60829 0.77544

C -0.76532 0.82854 1.71352

C -1.31758 -0.45812 -0.34941

C -2.59013 0.32205 -0.75151

O -0.55453 0.91091 2.90858

O -3.60941 -0.23579 -1.09552

N -2.48484 1.68116 -0.63715

N -1.20248 -0.32731 1.11368

C -1.76139 -1.40002 1.92526

H -2.82908 -1.52396 1.70766

H -1.25199 -2.34657 1.72800

H -1.63262 -1.11698 2.96883

C -0.08970 0.08550 -1.07364

H -0.27925 0.74131 -1.92289

C 1.20560 -0.31741 -0.85789

C 2.25021 0.24827 -1.66796

C 1.56566 -1.27112 0.15320

C 3.55305 -0.08919 -1.46879

H 1.98304 0.96596 -2.43875

C 2.87059 -1.62504 0.35031

H 0.78043 -1.72944 0.74255

C 3.88219 -1.02914 -0.45220

H 4.36236 0.32804 -2.05701

H 3.12851 -2.35328 1.10912

O -1.35707 -1.76428 -0.74266

H -2.32178 -2.19738 -0.72143

N -3.61951 -3.03629 -0.70630

H -4.34585 -2.40259 -0.37735

H -3.83776 -3.21169 -1.68631

C -3.61032 -4.29396 0.05453

H -3.39471 -4.07487 1.10265

H -4.55915 -4.83643 -0.00003

H -2.81530 -4.93604 -0.32918

H -3.30308 2.19595 -0.94669

O 5.15891 -1.29197 -0.32953

C 5.62123 -2.21957 0.66165

H 5.35722 -1.86474 1.66090

H 5.19900 -3.21023 0.47626

H 6.70200 -2.24576 0.54612

SCF energy: -1202.091744 hartree

zero-point correction: +0.396114 hartree

enthalpy correction: +0.422154 hartree

free energy correction: +0.340058 hartree

quasiharmonic free energy correction: +0.345272 hartree

**TS_MA_2 (MA)**

C -0.61968 4.41689 -0.45034

C -1.68958 3.58804 -0.71259

C -1.70269 2.27776 -0.20496

C -0.62397 1.81448 0.60754

C 0.44845 2.71844 0.86739

C 0.47518 3.97499 0.32711

H -0.61688 5.42295 -0.85830

H -2.51481 3.92684 -1.33154

H 1.23702 2.37498 1.53030

H 1.30495 4.64445 0.52338

C -0.83410 0.73849 1.67242

C -1.26761 -0.53780 -0.31405

C -2.62549 0.05406 -0.74754

O -0.62237 0.96622 2.84278

O -3.54821 -0.60771 -1.15543

N -2.72801 1.42922 -0.58330

N -1.25419 -0.44190 1.15116

C -1.74385 -1.53779 1.96417

H -2.81034 -1.70961 1.77607

H -1.18948 -2.45267 1.73852

H -1.59857 -1.26023 3.00843

C -0.10543 0.32591 -0.83582

H -0.31990 0.91300 -1.72796

C 1.25069 -0.05115 -0.67432

C 2.23358 0.61893 -1.45180

C 1.67662 -1.04817 0.23714

C 3.56259 0.31265 -1.32973

H 1.91946 1.38830 -2.15260

C 3.00942 -1.37375 0.35936

H 0.94063 -1.58155 0.82714

C 3.96696 -0.69129 -0.42017

H 4.32716 0.80946 -1.91607

H 3.31147 -2.15098 1.05020

O -1.07563 -1.79847 -0.77249

H -1.94474 -2.38679 -0.78261

N -3.10286 -3.43972 -0.84513

H -3.96266 -2.92481 -0.66376

H -3.15211 -3.72109 -1.82363

C -2.99226 -4.62451 0.01877

H -2.99274 -4.30625 1.06358

H -3.80656 -5.34119 -0.12569

H -2.04345 -5.12489 -0.18417

H -3.57651 1.83511 -0.96760

O 5.27027 -0.92313 -0.37172

C 5.77196 -1.93359 0.50099

H 5.53706 -1.69383 1.54207

H 5.35920 -2.91059 0.23279

H 6.85004 -1.93465 0.35582

One imaginary frequency: -226.57 cm^-1^.

SCF energy: -1202.084697 hartree

zero-point correction: +0.395251 hartree

enthalpy correction: +0.420769 hartree

free energy correction: +0.339613 hartree

quasiharmonic free energy correction: +0.344840 hartree

**Int_MA_2 (MA)**

C 1.49305 -4.28981 0.05998

C 2.40931 -3.34477 -0.33784

C 2.00054 -2.00343 -0.41759

C 0.64705 -1.59703 0.01449

C -0.29030 -2.69362 0.28508

C 0.12856 -3.97726 0.35431

H 1.81511 -5.32544 0.12719

H 3.42479 -3.62321 -0.60024

H -1.31711 -2.41366 0.50187

H -0.55342 -4.77756 0.61615

C 0.97118 -0.84337 1.44329

C 1.13907 0.70662 -0.35755

C 2.53082 0.35024 -0.92203

O 0.98546 -1.39582 2.50482

O 3.35258 1.11819 -1.33706

N 2.83533 -1.03969 -0.85113

N 1.24420 0.42432 1.09717

C 1.71374 1.43024 2.03327

H 2.76175 1.68196 1.83453

H 1.09816 2.32758 1.93324

H 1.62324 1.02263 3.04059

C 0.14696 -0.37630 -0.83441

H 0.33068 -0.59415 -1.89290

C -1.31339 -0.06102 -0.65358

C -2.22234 -0.63442 -1.55559

C -1.81939 0.71601 0.38821

C -3.58458 -0.44872 -1.41848

H -1.85215 -1.23723 -2.38247

C -3.18917 0.91776 0.53726

H -1.14729 1.20048 1.08813

C -4.08177 0.33102 -0.36446

H -4.29121 -0.88448 -2.11626

H -3.54547 1.53624 1.35206

O 0.75461 1.96049 -0.64534

H 1.52467 2.65592 -0.61224

N 2.53932 3.88771 -0.62922

H 3.49647 3.54021 -0.62554

H 2.39971 4.29849 -1.55183

C 2.34438 4.91259 0.40866

H 2.54923 4.47400 1.38789

H 2.98987 5.78680 0.27893

H 1.30270 5.23883 0.39267

H 3.78406 -1.28125 -1.13150

O -5.42032 0.45640 -0.30850

C -5.97415 1.24374 0.73081

H -5.72640 0.82777 1.71406

H -5.62276 2.27964 0.66905

H -7.05270 1.21518 0.58454

SCF energy: -1202.107282 hartree

zero-point correction: +0.397220 hartree

enthalpy correction: +0.422800 hartree

free energy correction: +0.342249 hartree

quasiharmonic free energy correction: +0.346751 hartree

**TS_MA_3 (MA)**

C 1.82218 -4.05598 -0.34722

C 2.66052 -2.97475 -0.57007

C 2.10315 -1.70123 -0.69597

C 0.69847 -1.51334 -0.60539

C -0.12707 -2.64130 -0.44636

C 0.42968 -3.89555 -0.28704

H 2.25547 -5.04288 -0.22099

H 3.73713 -3.10604 -0.62092

H -1.20240 -2.49702 -0.41275

H -0.20651 -4.75793 -0.12216

C 1.15623 -1.14529 1.60549

C 1.08926 0.80928 -0.01053

C 2.54264 0.69864 -0.53180

O 1.19447 -2.12305 2.22825

O 3.28964 1.64867 -0.59226

N 2.91693 -0.57804 -0.87097

N 1.20076 0.11892 1.42220

C 1.47829 1.01578 2.56288

H 2.45803 1.47620 2.41311

H 0.70138 1.78036 2.57882

H 1.47204 0.43883 3.48744

C 0.17686 -0.10598 -0.83466

H 0.39898 0.15847 -1.88047

C -1.30933 0.06459 -0.62063

C -2.16295 -0.23326 -1.69211

C -1.88272 0.44446 0.58965

C -3.53704 -0.16362 -1.55701

H -1.73906 -0.52694 -2.65025

C -3.26571 0.52794 0.74224

H -1.25909 0.71401 1.43579

C -4.10214 0.21915 -0.33305

H -4.20086 -0.38937 -2.38449

H -3.67490 0.84158 1.69498

O 0.65572 2.04677 0.13753

H 1.38790 2.83959 0.22928

N 2.15221 4.10225 0.20772

H 1.83251 4.82216 0.85370

H 3.12950 3.90777 0.41649

C 2.02659 4.56914 -1.18661

H 0.96972 4.72233 -1.41172

H 2.57284 5.49738 -1.37319

H 2.41465 3.78650 -1.84024

H 3.89536 -0.70479 -1.11083

O -5.44838 0.26434 -0.29583

C -6.06820 0.66965 0.91025

H -5.82104 -0.01410 1.73050

H -5.77468 1.69043 1.18043

H -7.14009 0.63769 0.72106

One imaginary frequency: -126.67 cm^-1^.

SCF energy: -1202.098863 hartree

zero-point correction: +0.395098 hartree

enthalpy correction: +0.420407 hartree

free energy correction: +0.340688 hartree

quasiharmonic free energy correction: +0.344864 hartree

**4'-Methoxyviridicatin 3 tautomer (MA)**

C -4.41258 -1.82931 -0.49537

C -3.85065 -0.70480 -1.08779

C -2.72003 -0.12711 -0.51743

C -2.13920 -0.65417 0.63848

C -2.71817 -1.78066 1.21711

C -3.84941 -2.36780 0.65925

H -5.29423 -2.28139 -0.93648

H -4.28801 -0.27538 -1.98477

H -2.26838 -2.20182 2.11204

H -4.28969 -3.24310 1.12380

C -0.71050 1.39451 0.80802

C -1.20549 1.80228 -0.59226

O -0.76288 2.80900 -1.14933

N -2.14544 1.01646 -1.13051

C -0.87327 -0.05065 1.19281

H -0.85239 -0.11674 2.28491

O -0.11547 2.22486 1.45889

C 0.41160 -0.68122 0.62542

C 1.61437 -0.47081 1.29978

C 0.42663 -1.40461 -0.57200

C 2.81366 -0.98674 0.81667

H 1.61586 0.09804 2.22730

C 1.61337 -1.91900 -1.06688

H -0.49841 -1.59382 -1.10938

C 2.81565 -1.71936 -0.37648

H 3.72720 -0.83059 1.37832

H 1.63828 -2.49684 -1.98432

N 1.21233 4.15876 0.05464

H 1.03480 4.19075 1.06243

H 0.33742 3.73908 -0.37806

C 2.35854 3.25633 -0.26177

H 3.27414 3.66044 0.16725

H 2.13888 2.27576 0.16240

H 2.44485 3.18211 -1.34494

H 1.36160 5.10845 -0.29492

H -2.50042 1.30791 -2.03719

O 3.91200 -2.26714 -0.93446

C 5.14034 -2.16357 -0.23730

H 5.87120 -2.70357 -0.83712

H 5.45164 -1.11710 -0.13774

H 5.06903 -2.62233 0.75510

SCF energy: -994.169401 hartree

zero-point correction: +0.343979 hartree

enthalpy correction: +0.365331 hartree

free energy correction: +0.293606 hartree

quasiharmonic free energy correction: +0.297751 hartree

**4'-Methoxyviridicatin 3 (MA)**

C 1.31943 4.34729 -0.14983

C 2.23640 3.31719 -0.10714

C 1.77861 1.99434 -0.07962

C 0.40558 1.68167 -0.07610

C -0.50203 2.76023 -0.14863

C -0.05510 4.06480 -0.18146

H 1.66557 5.37500 -0.17272

H 3.30401 3.52028 -0.10214

H -1.56399 2.54848 -0.19511

H -0.77105 4.87717 -0.24146

C 0.95176 -0.67857 -0.09111

C 2.36008 -0.35740 -0.09036

O 3.21789 -1.28762 -0.08131

N 2.69366 0.94712 -0.07164

C -0.01126 0.29215 -0.04236

O 0.65702 -1.99572 -0.08825

C -1.43927 -0.08305 0.05168

C -2.25294 0.44899 1.06526

C -2.00130 -0.99240 -0.84482

C -3.58330 0.09045 1.16669

H -1.82882 1.13920 1.78973

C -3.34308 -1.35099 -0.76356

H -1.38307 -1.42485 -1.62519

C -4.14212 -0.80757 0.24599

H -4.22031 0.48670 1.94995

H -3.75018 -2.05035 -1.48366

H 1.50637 -2.46927 -0.06527

N 5.59015 -2.21721 0.13960

H 4.70618 -1.59891 0.04570

H 6.17296 -2.10580 -0.69421

C 5.12634 -3.63011 0.29575

H 4.49913 -3.67888 1.18438

H 4.53679 -3.88609 -0.58295

H 5.98317 -4.29523 0.39148

H 6.14113 -1.91403 0.94660

H 3.68155 1.17376 -0.06866

O -5.45040 -1.08351 0.42023

C -6.06904 -1.97504 -0.48778

H -6.01386 -1.59408 -1.51391

H -5.60938 -2.96885 -0.43957

H -7.11154 -2.04063 -0.17952

SCF energy: -994.182687 hartree

zero-point correction: +0.343448 hartree

enthalpy correction: +0.364992 hartree

free energy correction: +0.292176 hartree

quasiharmonic free energy correction: +0.296962 hartree
