# Supplementary material for: Enzymatic one-step ring contraction for quinolone biosynthesis
Source: Nat Commun. 2018 Jul 19;9:2826. doi: 10.1038/s41467-018-05221-5 (PMC6053404; doi:10.1038/s41467-018-05221-5)
Supplement: Supplementary file 7 — Supplementary Data 4 [file 41467_2018_5221_MOESM7_ESM.docx]

**Supplementary Data Set 4.**

**(–)-Cyclopenin 5 (Zn + 2 His)**

C -4.65291 -2.18473 -2.51706

C -3.26796 -2.29998 -2.46616

C -2.57916 -2.02806 -1.27746

C -3.28283 -1.66819 -0.11064

C -4.68211 -1.56715 -0.18812

C -5.36397 -1.80419 -1.37546

H -5.17407 -2.39303 -3.44650

H -2.70825 -2.59735 -3.34864

H -5.22464 -1.31589 0.71671

H -6.44488 -1.71108 -1.40775

C -2.69048 -1.54476 1.26222

C -0.73628 -0.42189 0.29201

C -0.24590 -1.41539 -0.73911

O -3.29913 -1.93661 2.24976

O 0.98023 -1.50289 -1.00512

N -1.15884 -2.17495 -1.33570

N -1.42428 -1.00404 1.39793

C -0.77349 -1.12607 2.71147

H 0.23338 -0.71442 2.65428

H -1.34285 -0.57908 3.46667

H -0.72491 -2.17779 3.00591

C -0.89113 0.99473 -0.16973

H -0.64022 1.16063 -1.21652

C -1.79360 2.00200 0.43199

C -2.59760 2.74384 -0.44677

C -1.88142 2.22901 1.81310

C -3.49500 3.68789 0.05013

H -2.52299 2.57675 -1.51854

C -2.77313 3.18281 2.30359

H -1.24598 1.68241 2.50137

C -3.58414 3.90839 1.42665

H -4.11737 4.25398 -0.63674

H -2.83421 3.36014 3.37344

H -4.27923 4.64728 1.81494

O 0.26936 0.52692 0.61277

H -0.80134 -2.81901 -2.03971

Zn 2.35845 -0.11151 -0.30077

N 2.69005 1.84565 -0.90295

C 2.62128 2.95041 -0.15948

C 3.10295 2.23776 -2.16843

H 2.32270 3.00634 0.87624

C 3.27478 3.59332 -2.17628

H 3.24029 1.52776 -2.97055

H 3.58097 4.27860 -2.95125

N 3.80347 -1.40737 0.38264

C 3.73238 -2.79295 0.38526

C 4.97439 -1.07947 0.93087

C 4.87564 -3.29158 0.94281

H 2.87891 -3.31996 -0.01277

H 5.35201 -0.07972 1.08268

H 5.20923 -4.30148 1.12442

N 5.63832 -2.19380 1.27852

N 2.96595 4.01575 -0.90213

H 2.98642 4.97619 -0.57592

H 6.55528 -2.21849 1.71162

SCF energy: -3222.783678 hartree

zero-point correction: +0.433097 hartree

enthalpy correction: +0.462983 hartree

free energy correction: +0.371314 hartree

quasiharmonic free energy correction: +0.378401 hartree

**TS1 (Zn + 2 His)**

C -4.73970 -2.10878 -2.15940

C -3.38192 -2.36431 -2.27994

C -2.50951 -1.98021 -1.25445

C -3.00855 -1.35990 -0.05860

C -4.41703 -1.12528 0.01589

C -5.25907 -1.45823 -1.01844

H -5.40816 -2.40302 -2.96284

H -2.98296 -2.83886 -3.17191

H -4.81003 -0.69551 0.93106

H -6.32328 -1.25991 -0.94618

C -2.30241 -1.56790 1.28518

C -0.61789 -0.28338 0.12237

C -0.17849 -1.33820 -0.93559

O -2.87252 -2.13717 2.20537

O 1.01135 -1.44176 -1.26791

N -1.13733 -2.14256 -1.46075

N -1.04177 -1.06775 1.31462

C -0.13406 -1.29059 2.43051

H 0.71188 -1.92078 2.13058

H 0.24541 -0.33765 2.80716

H -0.69407 -1.79782 3.21696

C -1.85835 0.46904 -0.40750

H -1.95158 0.49411 -1.49140

C -2.45333 1.59421 0.25134

C -3.26060 2.45540 -0.53647

C -2.27628 1.88793 1.62889

C -3.86707 3.56840 0.02675

H -3.39772 2.23395 -1.59139

C -2.88285 3.00513 2.18181

H -1.66080 1.24588 2.24638

C -3.68073 3.84215 1.38723

H -4.48163 4.22254 -0.58368

H -2.74237 3.23180 3.23409

H -4.15728 4.71093 1.83226

O 0.36982 0.58709 0.36444

H -0.84734 -2.77113 -2.20878

Zn 2.23543 0.09310 -0.17215

N 3.57211 -1.40148 0.36433

C 3.64545 -2.64394 -0.10969

C 4.60825 -1.26132 1.27583

H 2.98287 -3.08137 -0.83981

C 5.30279 -2.43610 1.34579

H 4.77691 -0.33818 1.81090

H 6.16096 -2.73605 1.92704

N 2.99411 1.97422 -0.67593

C 2.24208 3.13811 -0.64753

C 4.20511 2.30849 -1.11553

C 3.01925 4.17828 -1.07501

H 1.21283 3.13264 -0.32357

H 5.04054 1.63955 -1.25670

H 2.81628 5.23099 -1.19710

N 4.67708 -3.28950 0.46326

N 4.25068 3.62996 -1.36336

H 5.05819 4.13572 -1.70993

H 4.94440 -4.25034 0.28038

One imaginary frequency: -225.13 cm^-1^.

SCF energy: -3222.758726 hartree

zero-point correction: +0.429865 hartree

enthalpy correction: +0.459897 hartree

free energy correction: +0.367551 hartree

quasiharmonic free energy correction: +0.375019 hartree

**Int (Zn + 2 His)**

C -4.88608 -2.29002 -1.76837

C -3.55068 -2.59572 -1.98568

C -2.57539 -1.86260 -1.31033

C -2.93521 -0.84110 -0.29264

C -4.36887 -0.52248 -0.21558

C -5.30038 -1.23900 -0.90114

H -5.64317 -2.86053 -2.29901

H -3.26235 -3.37097 -2.68845

H -4.66058 0.27545 0.45884

H -6.35758 -1.01815 -0.80216

C -2.44373 -1.52072 1.12685

C -0.61827 -0.32773 0.12408

C -0.22510 -1.34060 -0.99120

O -3.14912 -2.20726 1.83008

O 0.93940 -1.50131 -1.35468

N -1.24780 -2.06729 -1.56772

N -1.15450 -1.17278 1.25117

C -0.29564 -1.65920 2.32223

H 0.41925 -2.39985 1.94493

H 0.24910 -0.81942 2.75823

H -0.92548 -2.12488 3.08139

C -1.91985 0.37595 -0.39374

H -1.79733 0.60994 -1.45518

C -2.32454 1.65283 0.30589

C -2.70504 2.74052 -0.49752

C -2.35954 1.80520 1.70223

C -3.11506 3.94676 0.07090

H -2.68283 2.63880 -1.58010

C -2.76919 3.01241 2.27002

H -2.06105 0.99302 2.35548

C -3.15061 4.08478 1.45955

H -3.40668 4.77376 -0.57058

H -2.79004 3.11437 3.35131

H -3.47272 5.02041 1.90788

O 0.40925 0.43942 0.46773

H -0.98671 -2.76509 -2.26485

Zn 2.22653 0.02521 -0.19405

N 3.53933 -1.47583 0.35135

C 3.61306 -2.73256 -0.08228

C 4.53510 -1.32517 1.30584

H 2.97969 -3.18382 -0.82984

C 5.20525 -2.50824 1.44173

H 4.69305 -0.39014 1.82337

H 6.03072 -2.80417 2.07035

N 2.95628 1.91475 -0.70484

C 2.17710 3.05997 -0.64375

C 4.15977 2.28870 -1.13265

C 2.93033 4.12878 -1.04203

H 1.14883 3.02193 -0.31760

H 5.01148 1.64489 -1.29217

H 2.70344 5.17967 -1.13366

N 4.60627 -3.37716 0.55591

N 4.17407 3.61709 -1.34462

H 4.97038 4.15119 -1.67394

H 4.86829 -4.34562 0.40896

SCF energy: -3222.783959 hartree

zero-point correction: +0.432204 hartree

enthalpy correction: +0.462067 hartree

free energy correction: +0.370771 hartree

quasiharmonic free energy correction: +0.377664 hartree

**TS2 (Zn + 2 His)**

C -4.68437 -2.49875 -1.82662

C -3.32421 -2.64644 -2.07825

C -2.43280 -1.72432 -1.53003

C -2.89999 -0.65117 -0.69097

C -4.30691 -0.48893 -0.54413

C -5.17971 -1.42060 -1.06603

H -5.37791 -3.22704 -2.23600

H -2.95412 -3.46924 -2.68236

H -4.67456 0.35921 0.02306

H -6.24817 -1.31872 -0.90801

C -2.42491 -1.82386 0.97684

C -0.63802 -0.29516 0.13278

C -0.12246 -1.19642 -1.03200

O -3.23026 -2.59328 1.37077

O 1.09206 -1.29939 -1.26439

N -1.05899 -1.84502 -1.76315

N -1.24647 -1.32203 1.16952

C -0.44360 -1.73780 2.33416

H 0.21377 -2.56981 2.06403

H 0.14343 -0.87210 2.64160

H -1.11085 -2.04185 3.14217

C -1.88821 0.45980 -0.40427

H -1.58747 0.86402 -1.38084

C -2.33751 1.63920 0.43779

C -2.31502 2.91146 -0.15365

C -2.75494 1.53280 1.77458

C -2.68660 4.04860 0.56607

H -2.00366 3.01183 -1.19080

C -3.12646 2.66894 2.49521

H -2.79555 0.56883 2.27110

C -3.09200 3.93022 1.89600

H -2.66155 5.02265 0.08541

H -3.44507 2.56589 3.52881

H -3.38235 4.81171 2.46078

O 0.32761 0.41096 0.67151

H -0.74021 -2.49929 -2.47621

Zn 2.20772 0.06230 0.04402

N 3.67767 -1.30298 0.57234

C 3.84270 -2.51670 0.04872

C 4.68068 -1.13488 1.51441

H 3.22852 -2.96578 -0.71677

C 5.45124 -2.26341 1.54938

H 4.77584 -0.22669 2.09103

H 6.31627 -2.53090 2.13620

N 2.68304 1.88082 -0.82546

C 1.87461 3.00551 -0.76792

C 3.77035 2.22248 -1.51348

C 2.49127 4.02824 -1.43316

H 0.92535 2.98961 -0.25472

H 4.60822 1.58270 -1.74588

H 2.20304 5.05216 -1.61413

N 4.90256 -3.11755 0.61756

N 3.68239 3.50966 -1.89406

H 4.38063 4.01286 -2.43005

H 5.23930 -4.04857 0.39799

One imaginary frequency: -212.30 cm^-1^.

SCF energy: -3222.780639 hartree

zero-point correction: +0.430527 hartree

enthalpy correction: +0.460390 hartree

free energy correction: +0.369653 hartree

quasiharmonic free energy correction: +0.375535 hartree

**Viridicatin 6 tautomer (Zn + 2 His)**

C 4.91115 -2.61490 -1.43069

C 3.65810 -2.29463 -1.94572

C 2.76744 -1.55538 -1.16214

C 3.10641 -1.12296 0.12812

C 4.36838 -1.45723 0.62461

C 5.26596 -2.19917 -0.14427

H 5.60676 -3.19097 -2.03271

H 3.36468 -2.61625 -2.94136

H 4.64698 -1.13082 1.62270

H 6.24026 -2.45363 0.26119

C 0.73372 -0.35576 0.44142

C 0.47370 -0.71676 -1.01809

O -0.67327 -0.53107 -1.49153

N 1.48510 -1.25373 -1.69308

C 2.14436 -0.28833 0.95029

H 2.13752 -0.63031 1.99030

O -0.26077 -0.07703 1.11488

C 2.48421 1.22516 0.96781

C 2.33005 1.93662 2.16447

C 2.90146 1.89439 -0.19076

C 2.62043 3.30070 2.20808

H 2.00065 1.42036 3.06219

C 3.18522 3.26053 -0.14273

H 3.02854 1.35092 -1.12276

C 3.04728 3.96511 1.05506

H 2.52121 3.84107 3.14526

H 3.52015 3.77108 -1.04126

H 3.27688 5.02623 1.09169

H 1.29919 -1.52887 -2.65767

Zn -2.13431 -0.09954 -0.03046

N -2.85657 1.72132 -0.57712

C -2.47746 2.40845 -1.72206

C -3.75561 2.48060 0.05031

C -3.16110 3.58983 -1.77266

H -1.75300 2.00517 -2.41335

H -4.25827 2.24823 0.97686

H -3.15563 4.39760 -2.48826

N -2.91097 -1.89812 0.55462

C -2.61001 -2.58991 1.71982

C -3.84256 -2.60663 -0.08416

C -3.37077 -3.72384 1.76829

H -1.88183 -2.22618 2.42941

H -4.30523 -2.35566 -1.02674

H -3.43614 -4.51906 2.49486

N -3.95685 3.60994 -0.64770

N -4.13644 -3.70979 0.62304

H -4.59143 4.35574 -0.38305

H -4.81189 -4.41623 0.35128

SCF energy: -3014.831960 hartree

zero-point correction: +0.377833 hartree

enthalpy correction: +0.403475 hartree

free energy correction: +0.321530 hartree

quasiharmonic free energy correction: +0.326826 hartree

**Viridicatin 6 (Zn + 2 His)**

C -5.10676 -2.65974 -1.16843

C -3.76482 -2.93708 -1.36597

C -2.80013 -2.03362 -0.89367

C -3.17355 -0.83741 -0.22537

C -4.55343 -0.59626 -0.02694

C -5.50132 -1.48970 -0.49215

H -5.85678 -3.35440 -1.53390

H -3.44757 -3.84052 -1.87956

H -4.86409 0.29851 0.50047

H -6.55589 -1.28816 -0.33190

C -0.83191 -0.29293 0.00341

C -0.44625 -1.50652 -0.66340

O 0.77168 -1.81830 -0.87479

N -1.45101 -2.30581 -1.07545

C -2.13601 0.06280 0.22582

O 0.26275 0.47744 0.34142

C -2.42922 1.36084 0.89962

C -3.08305 2.39560 0.20777

C -2.01294 1.57765 2.22618

C -3.31747 3.61914 0.83432

H -3.40052 2.24004 -0.81926

C -2.25482 2.80507 2.84838

H -1.53352 0.77461 2.78110

C -2.90469 3.82647 2.15376

H -3.82281 4.41239 0.29100

H -1.94340 2.95589 3.87800

H -3.09350 4.77953 2.63943

H -0.02198 1.29526 0.79775

H -1.18730 -3.16344 -1.55643

N 2.62485 0.92240 -1.80987

C 1.72099 1.64356 -2.57568

C 3.84325 1.26406 -2.23081

C 2.41313 2.42329 -3.45883

H 0.65460 1.54945 -2.43981

H 4.78026 0.88658 -1.85119

H 2.08802 3.11948 -4.21604

H 4.52887 2.58487 -3.71103

N 3.74554 2.16644 -3.22064

Zn 2.20309 -0.46349 -0.36214

N 3.16562 -0.96221 1.37588

C 3.71050 -0.14184 2.27431

C 3.31009 -2.25142 1.86965

H 3.77240 0.93348 2.20515

C 3.94795 -2.19270 3.07634

H 2.94517 -3.11376 1.33134

H 4.65177 -0.46865 4.12238

H 4.24626 -2.95964 3.77393

N 4.18851 -0.85586 3.30722

SCF energy: -3014.855263 hartree

zero-point correction: +0.378134 hartree

enthalpy correction: +0.403759 hartree

free energy correction: +0.322073 hartree

quasiharmonic free energy correction: +0.327369 hartree

**Methyl isocyanate 7 (Zn + 2 His)**

O 1.90612 0.14289 0.00002

C 0.73699 -0.04952 0.00000

N -0.41484 -0.39227 -0.00006

C -1.74609 0.16538 0.00000

H -2.47217 -0.65070 -0.00497

H -1.91070 0.77498 0.89485

H -1.90761 0.78334 -0.88964

SCF energy: -207.966994 hartree

zero-point correction: +0.050934 hartree

enthalpy correction: +0.056531 hartree

free energy correction: +0.024116 hartree

quasiharmonic free energy correction: +0.024126 hartree
